# Sequence features of viral and human Internal Ribosome Entry Sites predictive of their activity

**DOI:** 10.1371/journal.pcbi.1005734

**Published:** 2017-09-18

**Authors:** Alexey A. Gritsenko, Shira Weingarten-Gabbay, Shani Elias-Kirma, Ronit Nir, Dick de Ridder, Eran Segal

**Affiliations:** 1 The Delft Bioinformatics Laboratory, Department of Intelligent Systems, Delft University of Technology, Delft, The Netherlands; 2 Platform Green Synthetic Biology, Delft, The Netherlands; 3 Kluyver Centre for Genomics of Industrial Fermentation, Delft, The Netherlands; 4 Department of Computer Science and Applied Mathematics, Weizmann Institute of Science, Rehovot, Israel; 5 Department of Molecular Cell Biology, Weizmann Institute of Science, Rehovot, Israel; 6 Bioinformatics Group, Wageningen University, Wageningen, The Netherlands; Tufts University, UNITED STATES

## Abstract

Translation of mRNAs through Internal Ribosome Entry Sites (IRESs) has emerged as a prominent mechanism of cellular and viral initiation. It supports cap-independent translation of select cellular genes under normal conditions, and in conditions when cap-dependent translation is inhibited. IRES structure and sequence are believed to be involved in this process. However due to the small number of IRESs known, there have been no systematic investigations of the determinants of IRES activity. With the recent discovery of thousands of novel IRESs in human and viruses, the next challenge is to decipher the sequence determinants of IRES activity. We present the first in-depth computational analysis of a large body of IRESs, exploring RNA sequence features predictive of IRES activity. We identified predictive *k*-mer features resembling IRES *trans*-acting factor (ITAF) binding motifs across human and viral IRESs, and found that their effect on expression depends on their sequence, number and position. Our results also suggest that the architecture of retroviral IRESs differs from that of other viruses, presumably due to their exposure to the nuclear environment. Finally, we measured IRES activity of synthetically designed sequences to confirm our prediction of increasing activity as a function of the number of short IRES elements.

## Author summary

Despite the importance of translation control in regulating gene expression across all kingdoms of life, for a long time no large collection of translation regulatory elements existed to facilitate in-depth computational analysis. In a recent study we devised a high-throughput reporter assay and employed it to discover and characterize thousands of ribosome recruiting sequences (Internal Ribosome Entry Sites, IRESs) in both the human genome and viruses. Here we use these sequences to perform the first in-depth computational analysis of a large body of IRESs, in which we explore RNA sequence features predictive of their activity. Our analyses provide insights on the effect of short RNA sequences on IRES activity, including their composition, number and position. We identified pyrimidine-rich sequence features resembling several known IRES *Trans*-Acting Factor (ITAF) binding motifs as predictive across human and viral IRESs, and discovered that their effect on IRES activity is strongest at distinct positions upstream of the start codon. Together, our results yield a high-level IRES architecture of sequence features and their spatial organization in RNA sequence, suggesting optimal positioning of ITAF binding sites, bringing us closer towards predicting protein levels from RNA sequence.

## Introduction

Translation of mRNA into protein is an essential step in the process of gene expression. Eukaryotic translation begins with the formation of the pre-initiation complex after the delivery of the ${\text{Met-tRNA}}_{\text{i}}^{\text{Met}}$ initiator tRNA to the P-site of the 40S ribosomal subunit by the eukaryotic initiation factor eIF2. The pre-initiation complex is then recruited to the 5′ untranslated region (5′-UTR) of the mRNA via the interaction between the 5′ m^7^GpppN cap structure, the poly-A tail of the mRNA, the poly-A binding protein (PABP) and additional initiation factors (eIF3 and eIF4) and begins scanning the 5′ UTR for the start AUG. Once the AUG is found in a favourable context, the 60S ribosomal subunit is assembled on the mRNA to begin protein synthesis [1, 2]. This translation initiation route accounts for more that 95% of cellular mRNAs [3], however, in a growing number cases alternative strategies are employed to initiate translation [4, 5]. One such strategy relies on the Internal Ribosome Entry Site (IRES) element, a *cis*-regulatory mRNA element that can attract the ribosome in a cap-independent manner. IRESs were first described as elements driving translation in poliovirus RNAs that do not possess the 5′ cap structure [6]. But IRESs were since discovered in other viruses, including HCV and HIV [7, 8, 9], in cellular genes such as p53 [10], XIAP [11] and Bcl-2 [12]. They were also shown to support the ongoing protein synthesis under conditions in which cap-dependent translation is inhibited, such as mitosis or cellular stress. The latter commonly occurs during viral infections, cancer and other human diseases [13, 14, 15]. Emerging evidence also suggests that in addition to this “back-up” mechanism, cellular IRESs also play important roles under conditions in which cap-dependent translation is intact: they facilitate the translation of different proteins from cellular bicistronic transcripts [16]; guide ribosomes to produce N-truncated isoforms from alternative downstream AUG codons [17, 18, 19]; and enable translation of transcripts with locally inhibited cap-dependent translation [20].

Despite this accumulating evidence of relevance of IRES elements to numerous diseases and cellular processes, compared to cap-dependent translation, relatively little is known about mechanisms of IRES-mediated translation. However, it is believed that a combination of primary sequence and RNA structure is functionally important for IRES activity [13, 22, 23, 24], which is achieved either via direct recruitment of the ribosome by the structured RNA, or through mediation by a combination of canonical initiation factors and additional IRES *trans*-acting factors (ITAFs; [24, 25, 26]). Precisely how ITAFs regulate IRES translation is not fully understood, but they are thought to function either as RNA chaperons, i.e. RNA-binding proteins (RBPs) that alter or stabilise RNA secondary structure in order to allow for ribosome binding, or as adaptor proteins interacting with the ribosome and other initiation factors [27]. Over a dozen proteins have been suggested to function as ITAFs [7, 25], but only few have been studied extensively. Among them, the PTB (polypyrimidine tract-binding protein) and PCBP (poly-C binding protein) RNA chaperon ITAFs were shown to remodel RNA structures of cellular IRESs [28, 29] for interactions with the 40S ribosomal subunit, and were proposed to have a similar role in viral IRESs [30, 31]. Whereas the hnRNP (heterologous nuclear nucleoproteins) C1/C2, the La autoantigen and Unr were implicated in modulating activity of multiple IRESs, but not in RNA structure remodelling [25].

Systematic methods to investigate mRNA translation have lagged behind the field of transcriptional control. Although isolated examples of IRESs with known ITAF binding sites or resolved three-dimensional structure are available [32, 33, 34], there are currently no systematic studies that aim at deciphering sequence elements governing cap-independent translation regulation. A major hindrance to progress in this direction is the relatively low number of known IRESs. The identification of novel IRES elements requires a series of labour-intensive reporter assays to confirm expression and to rule out the presence of cryptic promoter or splicing activity, so that only ≈120 IRESs were reported until recently [7]. Thus, unlike transcriptional regulation [35, 36, 37], attempts to systematically decipher determinants of cap-independent translation initiation were not feasible until now. In a recent work we developed a high-throughput IRES activity assay, and used it to identify thousands of novel IRESs in human and viral genomes [21], thereby expanding the dataset of known IRESs by 50-fold and allowing for the first time the construction and interpretation of predictive models.

Here we perform an in-depth computational analysis of data from our high-throughput IRES activity assay [21] to explore the relationship between RNA sequence and IRES activity. We find several common sequence *k*-mer features predictive of IRES activity that are shared between (i) sets of viral IRESs originating from viruses of the same type, and (ii) sets of cellular IRESs originating from similar locations within human transcripts, as well as features specific to retroviral IRESs. These features include the poly-U, poly-A and C/U-rich *k*-mers, many of which are found upstream of the start AUG in distinct “location islands”, continuous stretches of positions where these sequence features have the strongest effect, suggesting that positions of ITAF binding sites relative to the AUG are important determinants of IRES activity. Finally, systematic measurements of hundreds of fully designed synthetic oligos confirmed our finding of a positive relationship between the number of short IRES elements in a sequence and its IRES activity. Together, we provide the first in-depth computational analysis of thousands of IRESs from the human genome and different types of viruses and offer novel insights into the relationship between RNA sequence and IRES activity.

## Materials and methods

### Dataset

In a recent study [21] we described a high-throughput IRES activity assay that we used to measure IRES activity for thousands of sequences, including 28,669 native fragments from the human and viral genomes. In the current study we use these measurements to uncover RNA sequence and structure determinants of IRES activity. Detecting IRESs using bicistronic DNA constructs can be subjected to potential artifacts of cryptic promoters and splicing sites and lacking suitable controls in the past had led to controversy about the authenticity of newly discovered elements. Thus, a large portion of the original study was dedicated to rigorous controls showing that the detected IRESs are neither cryptic promoter artifacts nor splice site artifacts [38]. Among these are two additional high-throughput assays devised specifically to measure promoter and splicing activities; qRT-PCR experiments on the upstream cistron of the bicistronic construct with three sets of primers; qRT-PCR experiments on the two cistrons in isolated clones; and validation of selected IRESs in traditional mono-cistronic and bi-cistronic luciferase constructs. Due to the importance of this issue, we discuss these extensive controls as well as detailed examples of the high agreement between our measurements and established findings from previous studies (S2 Text and [39]).

**Fig 1:**
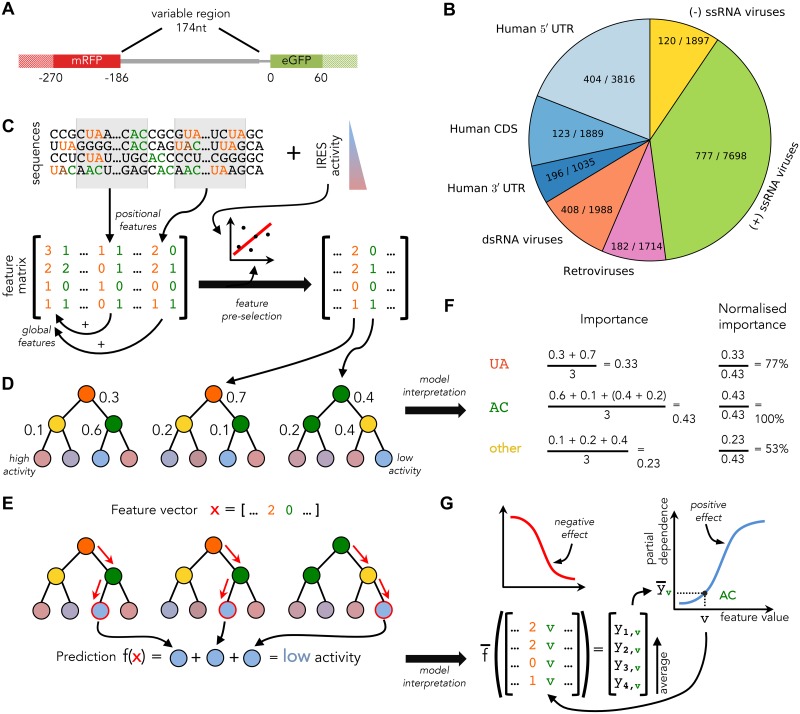
Overview of the available data and our analysis approach. (A) Schematic representation of the bicistronic reporter construct used in [21] with eGFP (green) expression used to measure IRES activity of variable sequences (gray), and constitutively expressed mRFP used to control for unique genomic integration. To capture context effects, in our analyses the assayed variable sequences (thick gray) were extended to include flanking regions (solid filling). (B) The available sequences can be divided into 7 groups based on their origin species and location within transcripts. Number of active sequences, i.e. sequences with IRES activity above background levels, and the total number of RNA sequences are shown for each class. (C) Sequences from each of the groups are represented as vectors of sequence *k*-mer features (UA—orange, AC—green), which are recorded globally and in windows (gray shading). From this large set of features, those unlikely to be predictive are removed based on their weak correlation with IRES activity. Surviving features are used to construct a reduced feature matrix. (D) The reduced feature matrix is used for Random Forest training. Each RF tree consists of decision nodes (coloured according to the variables selected by those nodes during training) and leaf nodes that predict IRES activity (coloured according to their prediction). RF trees are constructed by iteratively selecting for each node a variable and split that yield the highest reduction in weighted variance in the nodes children; normalised variance reduction is shown for every node as a number. (E) Trained RFs are used to make IRES activity predictions for feature vectors *x* of unseen sequences by following each tree to the leaf node corresponding to *x* (path and leaves marked in red), and accumulating leaf node predictions to obtain the overall RF prediction *f*(*x*). (F) To select features that are most predictive of IRES activity, variance reduction values from (D) are accumulated per tree and averaged across trees to obtain *feature importance*. Normalised importance is also calculated for use in model interpretation. (G) To understand the effect of a feature (e.g. the AC *k*-mer), for each of its possible values *v* the expected prediction ${\bar{y}}_{v}$ is plotted (blue curve). The resulting curve allows for characterising *v* either as having a positive (increasing curve, blue), or a negative (decreasing curve, red) effect on IRES activity. Expected predictions ${\bar{y}}_{v}$ are approximated as the average of predictions made for training samples with the corresponding feature vector components substituted by value *v*.

The library measured in [21] includes sequences originating from human transcripts and viral genomes. In particular, the library sequences were generated by (i) taking the sequences directly upstream of transcripts’ translation start site; and (ii) by tiling transcripts and viral genomes with sequences to be measured. Because most sequences in such library are not expected to have IRES activity, ≈11% of the sequences showed activity above background levels (see Fig 1B and S2 Fig). Library sequences were taken from genomes of viruses with considerably different life cycles and replication strategies. Differences in the available host gene expression machinery and subjection to distinct selection pressures due to the employed replication strategies [40, 41] may have prompted different viral classes to evolve distinct cap-independent translation strategies [42]. For this reason we separated viral sequences into (i) positive-sense (+) ssRNA viruses; (ii) negative-sense (−) ssRNA viruses; (iii) dsRNA viruses; and (iv) retroviruses based on their viral class (Baltimore classification) (Fig 1B). In the case of human transcripts, our measurements uncovered significant differences in IRES activity for different regions (S9 Fig). This observation, together with mechanistic differences between these regions [43, 44], led us to divide human sequences from the library into those originating from (i) the coding sequences (CDSes); (ii) the 5′ UTRs; and (iii) the 3′ UTRs (Fig 1B).

We analysed the above seven groups of sequences both together and individually. For each of the groups we learned a predictor of IRES activity from RNA features with the goal of elucidating sequence features that may determine IRES activity, and would consequently provide a prediction of the IRES activity for novel sequences.

### Random Forest model learning

Our approach for learning sequence models of IRES activity is depicted in Fig 1C–1E. We chose Stochastic Gradient Boosting Random Forest regression for learning sequence models for several reasons. First, Random Forests (RFs) allow for construction of nonlinear predictors that offer established model interpretation techniques. Second, stochastic gradient boosting allows for achieving highly accurate predictions by fitting the gradient of the residual error with every new tree added to the forest, while being fairly robust to overfitting in practice [45]. The latter is especially important in our case, because for some of the considered groups of IRES sequences only a few hundred training instances are available (sequences with measured IRES activity) while thousands of features (*M*) are being used, leading to a situation that can easily result overfitting.

We used the scikit-learn software [46] to learn RFs from training data. We chose to train 1000 trees per forest. To speed-up the training process, each tree only evaluated $\sqrt{M}$ features when choosing split features. The trees were allowed to have arbitrary depth, but their complexity was controlled by parameter *m*, defining the minimum allowed number of training samples per leaf node. This parameter was set, together with the learning rate *r* and subsampling fraction *f*, using a double-loop 10-fold cross-validation (CV) scheme on the available training data (described in detail in S2 Fig). Briefly, each outer CV training set was randomly partitioned into 10 sets; every time, 9 of these sets were used as an inner training set and the remaining set was used for validation. For each of the 10 inner training sets, we learned an RF for every combination of the parameters (*m*,*r*,*f*) from a pre-defined grid and evaluated its performance (in terms of the *R*^2^ statistic) on the held-out inner validation set. The parameter set with the highest average performance across the 10 validation sets was used for learning the final predictor on the outer CV training data, which was evaluated on the outer CV validation set. When randomly partitioning sequences into CV folds, we ensured that the numbers of sequences with background levels of IRES activity were balanced across sets.

### *k*-mer feature pre-selection

To explore the relationship between IRES sequence and activity, we described its primary sequence using numerical features which could be related to IRES activity by the learned RFs. We chose to represent IRES RNA sequences using *k*-mers, as they were previously successfully employed for modelling and understanding determinants of several transcriptional mechanisms [37, 47, 48, 49], and thus provide a promising starting point for modelling sequences determinants of IRES translation. To this end counted how many times every possible RNA subsequence of length *k* ≤ 5 occurs the training sequences (see example in Fig 1C). These counts were recorded for the entire sequences (global counts), as well as in moving windows of 20nt with a 10nt overlap (positional counts) to generate position-sensitive *k*-mer features. To assess the added predictive power of the *k*-mer copy numbers, we also created a *k*-mer occurrence feature description of the available RNA sequences, in which *k*-mer counts were capped at a maximum value of 1.

Because this representation of IRES sequences generates thousands of features, to facilitate model learning and interpretation we sought to reduce the number of used features by pre-selecting them prior to RF training. To this end, on the inner training set for each feature we (i) computed correlation coefficient and *p*-value for the Spearman rank correlation between feature values and IRES activity for *k*-mer counts; or, for *k*-mer occurrences, the Mann-Whitney U-test statistic and *p*-value to assess the difference between IRES activity distributions for sequences with and without the feature; and (ii) counted in how many training samples the feature value was non-zero. To keep the number of model input features manageable, only features with an association significant at a false discovery rate (FDR) of 0.05 (controlled using the Benjamini-Hochberg procedure) and present in at least 10% of the sequences were used for model learning. Together, these criteria implicitly control the FDR of the *k*-mers chosen for model interpretation to well below 0.05 (see the following section).

### Random Forest feature interpretation

Unlike linear models relying on *L*_1_ regularisation (e.g. [50, 51]), RFs cannot perform simultaneous feature selection and learning. This means that all features provided to RFs will generally be used by the learned model to make predictions. This property of RFs complicates model interpretation by increasing the number of features of the learned model that need to be examined. To efficiently sift through the features we calculate their *feature importances* as in [52] and use them to select and prioritise interesting features (see Fig 1D and 1F). For each tree in an RF, the feature importance of a variable captures its contribution to the resulting prediction by quantifying the total reduction in variance the variable provides each time it is selected as a split feature in this tree. The importance of a variable in an RF is then calculated as its average feature importance across all RF trees. To facilitate comparison of feature importances across models with different numbers of features, i.e. models obtained for different CV folds or sequence groups, we normalised importances of every model by dividing its feature importances by the maximum feature importance attained.

Similarly, because RFs do not provide a direct way of evaluating the direction of the effect (positive or negative) features have on the resulting prediction, we computed the *partial dependence* [52] of an RF w.r.t. its features at all possible values (see Fig 1E and 1G). Partial dependence of a feature provides an estimate of the expected prediction (IRES activity) of a sequence with a given value for this feature. When plotted for all possible values of a selected feature, partial dependence allows for graphic inspection of the relationship between the feature and IRES activity. We observed that in practice, partial dependence often shows near-monotonic behaviour (see S3 Fig for representative examples), i.e. the expected prediction either tends to increase (or to decrease) with increasing feature values, and used this property to determine directionality of each feature based on the average derivative of its partial dependence. Features were classified as increasing IRES activity (positive) if their average derivative was positive, otherwise they were classified as negative (decreasing IRES activity). This classification can be thought of as a generalisation of the linear model variable separation into positive and negative based on their slopes (i.e. model coefficients).

To obtain robust results, partial dependences and feature importances were averaged across 10 RFs models trained on different outer CV folds.

### Synthetic data design

We designed a total of 512 oligos in which we planted the sequence of the TEV IRES (UACUCCC) [53] in 1-8 copies. Each oligo is composed of 164nt of variable sequence, 10nt of unique barcode at the 5′ end (barcodes differ by at least 3nt from each other) and constant primer sequences to amplify the oligos with PCR reaction. We chose one native and one synthetic background sequence (see S1 Table), which lack intrinsic IRES activity: (i) 164nt of the human beta-globin gene (HBB, NM_000518) that was used as a negative control in a previous study [54], and (ii) a concatenation of a 9-mer that was used as a spacer between multiple copies of the Gtx IRES in a previous study (Spacer1: TTCTGACAT; [55]). This set of 512 sequences was measured for IRES activity as part of a 55,000 oligos library in a high-throughput bicistronic assay described before [21] and analysed here for the first time.

### Data availability

The source code and data used to produce the results and analyses presented in this manuscript are available from their Bitbucket Git repository https://bitbucket.org/alexeyg-com/irespredictor.

## Results

### Prediction of IRES activity from sequence

With the recent discovery of thousands of novel IRESs in human and viruses, providing a 50-fold increase over previously available data [21], the next big challenge is to uncover the RNA sequence features predictive of IRES activity. We sought to employ a machine learning approach for this purpose, in which we train Random Forests to predict IRES activity from RNA sequence features, and then use the trained forests to uncover predictive sequence features. To this end we computed *k*-mer and structural features for all 20,872 available native IRES sequences, randomly partitioned the sequences into 10 sets of near-equal size and used them in a cross-validation scheme to train and test 10 independent RF models (see Materials and methods). To get a comprehensive evaluation of model performance, we used five metrics to evaluate its ability to predict exact IRES activity levels, including the *R*^2^ statistic, which quantifies the portion of variance in the data that is explained by the models, the Pearson correlation, *r*, and the Spearman rank correlation, *ρ*, calculated on test set predictions. Although the model was trained to predict exact IRES activity levels (i.e. regression setting), we also used two additional metrics to evaluate its ability to separate positive sequences (measured activity above detection limit) from negative: the area under the receiver operating characteristic curve (AUC-ROC) and the area under the precision-recall curve (AUC-PR) (see S1 Text).

**Fig 2:**
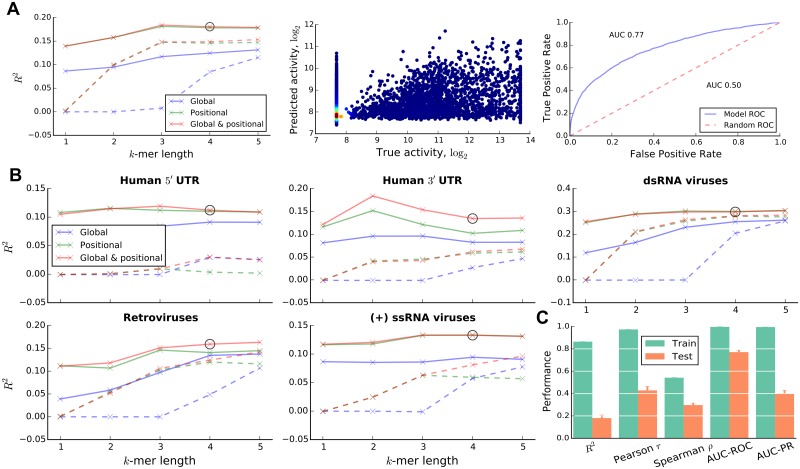
Performance of trained predictors. (A) Cross-validation (CV) performance of models trained on all available native IRES sequences shown for different combinations of *k*-mer lengths, and *k*-mer count (solid lines) or presence (dashed lines) features (left), with the selected combination marked with a circle. Scatter plot of predicted and true IRES activities for the selected model (middle) coloured according to the local density (blue to red as low to high density). The Receiver Operating Characteristic (ROC) curve and the area under the curve (AUC) for the selected combination. (B) CV performance of models trained for different groups of sequences. Only results for groups with models achieving sufficiently high performance are shown. (C) Training and test performance of the feature and *k*-mer length combination selected for the group of all native IRESs evaluated using several metrics.

In a previous study we found that the effect of mutations on expression was not uniform across the IRES sequence, suggesting that in addition to the sequence of the functional elements, their position within the IRES is also important [21]. Thus, we tested the effect of both, global sequence features (counts of *k*-mers within the examined sequence) and positional sequence features (counts of *k*-mers within a specific region of the examined sequence; Fig 1C). Further, we sought to check whether *k*-mer copy number information provides additional predictive power, compared to *k*-mer presence (*k*-mer counts capped at a maximum value of 1), and considered both feature representations in our models. We first learned combined models of IRES activity on the entire set of sequences without separation into groups based on virus type or location within transcripts. The models were learned for different combinations of *k*-mer length and *k*-mer feature types (global or positional; count or presence). The highest predictive power was achieved by a model that makes use of the global and positional 3-mer or 4-mer count features (see Fig 2A, left). We selected this model with *k* = 4 for further analysis. Its test set *R*^2^ is 0.18, indicating that RNA sequences can explain 18% of the variance in IRES activity of cellular and viral IRESs in human cells. The agreement between *R*^2^ and the Pearson *r* of 0.429 (Fig 2C) suggests that our models correctly capture the mean IRES activity in unseen test data. However, the differences between the test set Pearson and Spearman correlations (*r* = 0.429 and *ρ* = 0.297; Fig 2C) indicate that the models are biased towards better prediction of extreme IRES activity values, as can be seen from the bright red spot in the lower left corner of the scatter plot in Fig 2A (middle). This behaviour is expected for the skewed IRES activity distribution of the available sequences (see S1 Fig), in which the negative skew can be explained by the relatively low abundance of IRESs in human and viral genomes [56]; and by potential underestimation of IRES activity due to its dependence on cellular conditions.

The models ability to predict IRES activity also translates to its ability to separate positive and negative IRES sequences, as evident from the ROC curve in Fig 2A (right) and the AUC-ROC and AUC-PR measures in Fig 2C (see also S1 Text). Interestingly, the model appears to be better at separating the positive and negative sequences (AUC-ROC and AUC-PR of 0.77 and 0.40 respectively, compared to 0.50 and 0.11 for random predictions) than at predicting the exact activity levels, as also suggested by the widely scattered cloud of points in Fig 2A, middle. This result is unsurprising, however, since the task of predicting the exact activity levels is inherently more difficult. Given the good agreement between the considered evaluation metrics, we chose to use the *R*^2^ statistic in all our analyses.

We hypothesised that IRESs from different virus types and locations within human transcripts may have evolved distinct initiation mechanisms [42]. To capture these distinct mechanisms, we separated the available human data based on their location within transcripts into sequences from (i) human 5′ UTRs, (ii) human 3′ UTRs and (iii) human CDSes; and the available viral data based on their virus type into sequences from (iv) positive-sense ssRNA viruses, (v) negative-sense ssRNA viruses, (vi) dsRNA viruses and (vii) retroviruses, irrespective of their position in the viral genome of origin. Due to the reduction in the number of available training samples, the performance of models trained on these groups is expected to be lower than that for the group of all sequences, unless the individual groups consist of sequences with distinct IRES translation mechanisms that are easier to learn in isolation. We learned RF models for each of the groups as before. As can be seen from their test *R*^2^ in Fig 2B, in line with our expectation, for most sequence groups the models’ predictive power is reduced. Remarkably however, the *R*^2^ statistic for the group of dsRNA viruses is increased to 0.298, a considerable improvement in predictive power over the combined mode. This suggests that this sequence group is easier to model in isolation, presumably because the proposed division into groups achieves the goal of separating sequences with distinct IRES translation mechanisms from each other. At the same time we also found that in some groups IRES activity cannot be predicted by the proposed approach (e.g. the human CDSes, *R*^2^ ≈ 0, or the negative-sense ssRNA viruses, *R*^2^ = 0.036; see S4 Fig). Translation initiation of IRESs from these groups may rely on mechanisms that are poorly captured by primary sequence features, such as those involving pseudoknots and the three-dimensional structure of RNA molecules. Additionally, these groups have the lowest absolute and relative incidence of active IRESs (≈6.4%), which makes it difficult to learn predictive models (see S5 Fig). To further support our strategy of dividing sequences into groups, we ensured that the variation in predictive power between groups observed for the proposed division is unlikely to obtain by chance (*p* < 10^−3^, see S1 Text).

Interestingly, models based on the *k*-mer count features consistently achieved higher performance their *k*-mer presence counterparts across all sequence groups. While this result is unsurprising, given that the count features provide a richer description of the sequences than the capped presence features, it also suggests possibilities for a regulatory effect of *k*-mer copy number on IRES activity.

We have also considered several types of RNA structure features, which captured local RNA accessibility and base pairing between regions of the RNA. Individual structural features were pre-selected based on their correlations with IRES activity and used for model training in the same way as *k*-mer count features were (see S1 Text). However, despite being weakly predictive when used in isolation (*R*^2^ < 0.02; S1 Text), the considered types of structural features did not allow for increasing model predictive power beyond what could be achieved using *k*-mer features alone.

The difference between train and test performance (Fig 2C and S1 Text) suggests that the models were overfit on the training data. However, this does not diminish the models’ ability to predict IRES activity of unseen sequences, as measured by their CV test performance. Further, as discussed in the following section, the potential overfitting is not a big concern in light of the strict criteria used for selecting *k*-mer features for interpretation.

### C/U-rich *k*-mers are strong determinants of IRES activity

Having obtained several predictive models, we sought to use them to elucidate individual sequence features that are strong determinants of IRES activity. Given the superior performance of models trained on the combination of global and positional count features (Fig 2A and 2B), we chose to interpret them, as it would provide a more faithful view of IRES features. Additionally, we chose to interpret models with *k* = 4 for all sequence groups irrespective of whether the highest predictive power is achieved at this *k*-mer length. This choice facilitates feature comparison at the cost of a negligible drop in performance for some sequence groups. Further, only the 5 groups with useful predictive models (*R*^2^ > 0.1; Fig 2B) were analysed.

**Fig 3:**
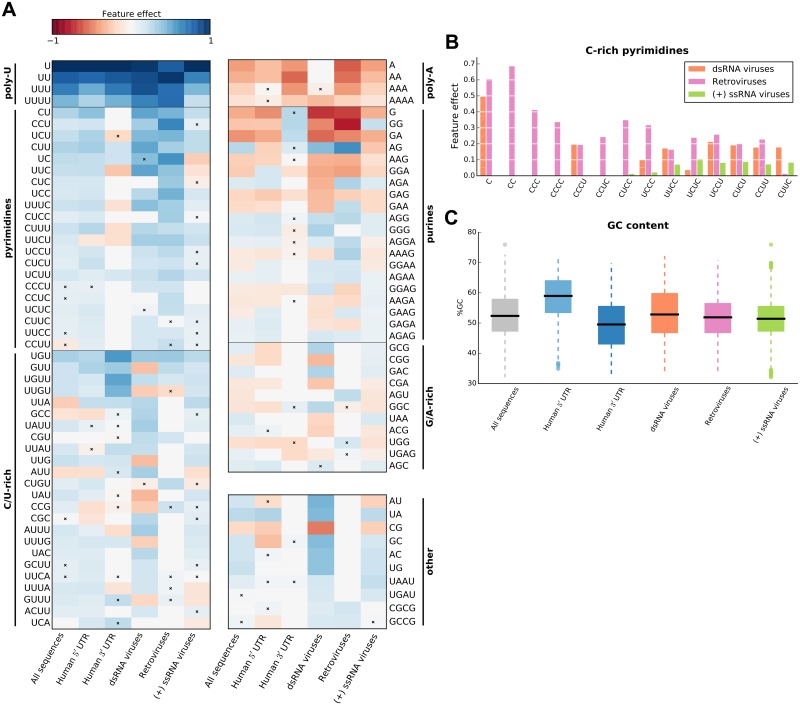
Overview of IRES global sequence features. (A) Robust and predictive global *k*-mer count features that appear in at least two IRES sequence groups; features were divided into classes based on their nucleotide composition and interpretation (vertical bars). For each feature, its effect (feature importance taken with sign “+” if the feature was classified as positive, and with sign “−” otherwise) is shown, and non-robust features are marked with a cross. (B) Comparison of C-rich pyrimidine tract feature importances across three viral sequence groups; non-robust features are shown with hatched bars. (C) Sequence GC content distribution for the defined sequence groups.

For every sequence group we took *k*-mer features that were robust (present in all 10 CV models) and predictive (defined as having an average feature importance of at least 0.1; see Fig 1D and 1F). Combined with the *k*-mer pre-selection strategy used prior to model training (see Materials and methods), these strict criteria minimise the chance that spurious *k*-mer features are identified as robust and predictive, and thus chosen for interpretation. For each of the selected features we also determined its directionality (positive or negative) from the shape of its partial dependence plot (see Materials and methods, and Fig 1E and 1G). We first sought to examine features that are consistently related to IRES activity across multiple sequence groups, i.e. common features, and thus focused on those *k*-mers that were predictive and robust in at least two groups. In Fig 3A we show common *k*-mer count features separated into several classes based on their composition and effect; the remaining non-common features are shown in S6 Fig.

Our predictive *k*-mer analysis recapitulates the findings from [21], as we also show that *k*-mers presenting the poly-U motif are consistently selected in all sequence groups with poly-U *k*-mer presence being associated with increased IRES activity. However, in addition to the poly-U motif discussed in [21], we found that (i) *k*-mers representing pyrimidine (C/U) tracts are also strong determinants of IRES activity; and that (ii) these *k*-mers can equally contribute to the activity of IRESs from various positions on the transcripts and in various types of viruses.

Poly-A *k*-mers represent another group of features shared across models for different sequence groups. However, adenine tracts were not previously associated with decreased IRES activity in human cells. Selection of these *k*-mers by the trained models may be a consequence of an anti-correlation between the count of A/G and U/C nucleotides in the measured sequences. However, Poly-G *k*-mer are generally not present in the trained models, suggesting that a mechanism specific to Poly-A tracts is involved in IRES-mediated translation. Similarly, the purine tract features, which are mostly associated with decreased IRES activity, can be explained by an anti-correlation between presence of purines and pyrimidines in sequences, and by an additional adenine tract specific mechanism.

Our results suggest that despite differences in model predictive power between sequence groups, robust and predictive global *k*-mer features are often shared by multiple groups, in which they agree on the effect they have on IRES activity (Fig 3A and S6 Fig). However, we also sought to uncover features that are specific to a single sequence group or viral class. When reviewing features that were robust and predictive only for a single sequence group (S6 Fig), we found that a number of pyrimidine tract features (*C*_1−4_ and *UC*_3_) were uniquely selected for the retroviruses group. Interestingly, these features are all C-rich *k*-mers, whereas the common pyrimidine tract features, shared by multiple sequence groups, are not (Fig 3A). This preference of retroviral IRESs for C-rich *k*-mers can be clearly seen from differences in feature importances of C-rich pyrimidine tract features across viral sequence groups (see Fig 3B), which show that C-rich features are either uniquely used by the retroviral predictive models, or have the highest importance in those models. Furthermore, preference for C-rich *k*-mers within the group of retroviral sequences does not appear to be a consequence of GC-content bias, which is similar between retrovirus and (+) ssRNA virus groups (Wilcoxon rank-sum test, *p* > 0.06) and lower in retroviruses compared to dsRNA viruses (Wilcoxon rank-sum test, *p* < 10^−7^; see Fig 3C).

### Systematic measurements reveal that increasing the number of a C/U-rich IRES element leads to elevated activity

**Fig 4:**
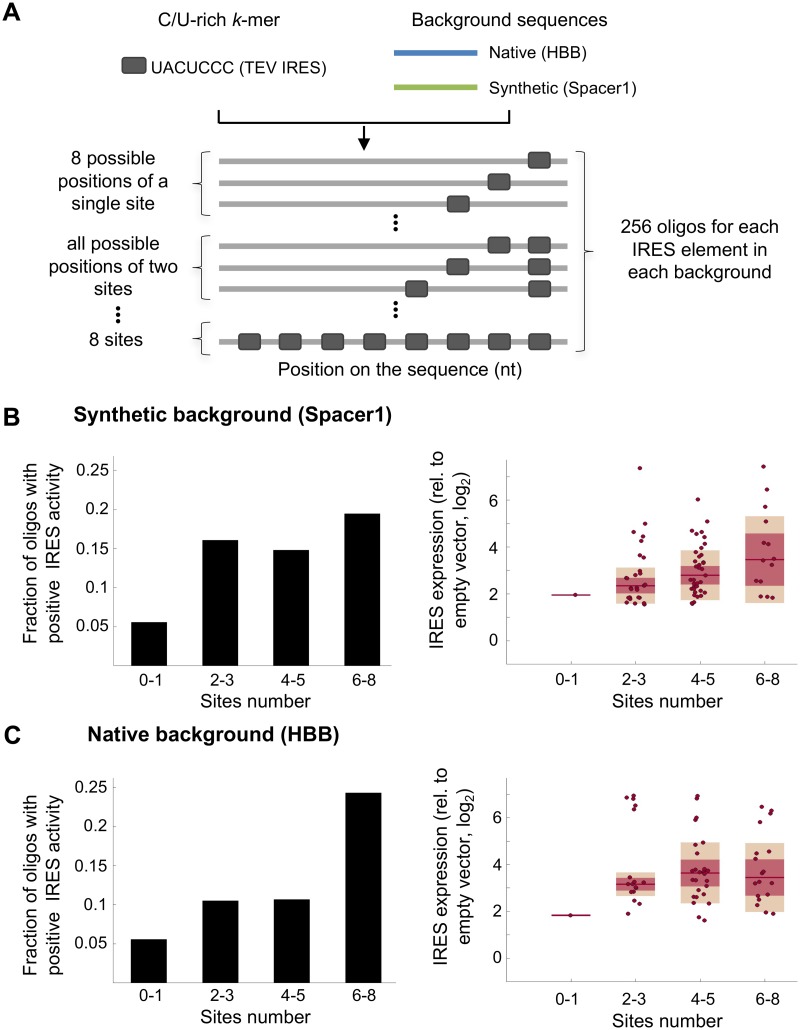
Testing the effect of the number of C/U-rich elements on IRES activity using synthetic oligos. (A) The TEV IRES element was placed in all possible combinations of 1-8 sites in predefined positions on two background sequences (native and synthetic; coloured lines) to generate synthetic oligos (gray blocks and lines), which were measured using the biscistronic IRES activity reporter assay. (B and C) Oligos were binned into four groups according to the number of placed elements: (left) the fraction of oligos with positive IRES activity from the total designed oligos is shown for each bin; (right) box plots showing the expression levels of oligos with positive IRES activity in each bin. Results are shown for a synthetic background (B) and a native background from the human beta-globin gene (HBB) (C).

Collectively our *k*-mer count feature analyses (Figs 2 and 3A and S3 Fig) suggest that increasing the copy number of short “IRES elements” in an mRNA sequence would lead to increased IRES activity. In order to systematically test the effect of the number of elements on expression we investigated the expression measurements of synthetically designed oligos, in which we planted the reported C/U-rich Tobacco Etch Virus (TEV) short IRES element UACUCCC [53] in 1-8 copies. To control for the effects of additional parameters varied between designed sequences, such as the distance of the site from the start AUG, the distance between two adjacent elements and the immediate flanking sequence in each position, we placed the TEV IRES element in all possible combinations of 1-8 sites at 8 predefined locations within two different backgrounds, resulting in a total of 512 oligos (256 oligos for each background; Fig 4A, S2 Table). We chose one synthetic background and one native background from the human beta-globin gene (HBB), both lacking intrinsic IRES activity [54, 55]. This set of sequences was measured for IRES activity as part of the 55,000 oligos library described before [21]. To test the relationship between the number of C/U-rich elements and IRES activity we binned the data into four groups according to sites number: 0-1, 2-3, 4-5 and 6-8. To increase the power, we performed joint analyses of two independent biological replicates. For each group we computed both, the fraction of designed sequences with positive IRES activity (threshold was defined according to empty vector measurements [21]), and the expression levels of the positive sequences. This analysis revealed that increasing the number of C/U-rich elements leads to higher fraction of positive IRESs and that these IRESs are more active in general in the two backgrounds tested (Fig 4B and 4C, S8A and S8B Fig). Together, elevating the number of sites results in higher IRES activity (*p* < 0.003, one-way ANOVA, S8C Fig).

### *k*-mer position is a strong determinant of IRES activity

Having obtained a rendering of the global *k*-mer features predictive of IRES activity, we sought to expand our analysis of the effect that *k*-mer location may have on IRES activity. We were encouraged by the results of training models on different combinations of global and positional *k*-mer features (Fig 2B) which showed that for all sequence groups models trained on positional features achieved highest performance, suggesting that *k*-mer position relative to the start AUG is a strong determinant of IRES activity.

**Fig 5:**
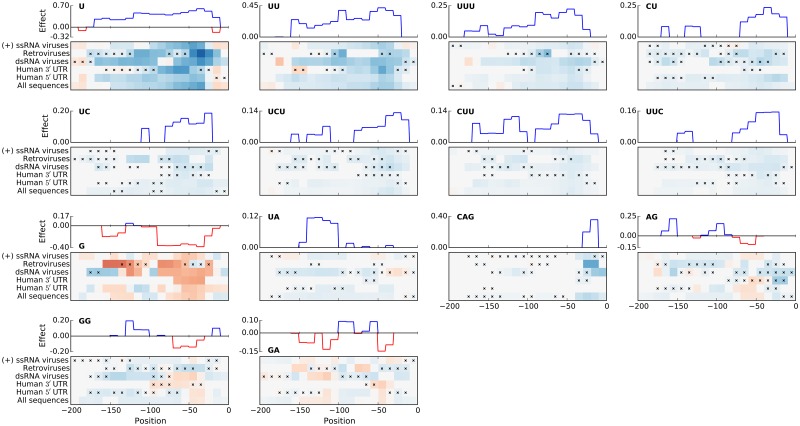
Robust and predictive positional features that appear in at least two of the analysed groups. For each feature, its effect along sequences is shown in a heat map (see Fig 3), and summarised as a consensus effect (located above each of the heat maps) across several groups, chosen as the effect whose directionality and importance are confirmed by at least two groups. Horizontal axes show feature window position relative to the start AUG.

To investigate this further we assessed the effect of positional *k*-mers as a function of their location in the sequence. We first focused on those positional *k*-mer features that were common to multiple sequence groups. To this end positional features were investigated only for those *k*-mers, which showed a robust location-specific signal (had at least two windows where the *k*-mer feature was selected in all CV folds), were predictive (had an average importance in those windows of at least 0.1) and were shared by several sequence groups (i.e. the windows were also robust and predictive for at least one more group). Common positional features in Fig 5 are shown as heat maps depicting *k*-mer effect along the sequence and across sequence groups, which is summarised as a consensus effect, i.e. the largest effect at a particular position that is supported by multiple groups; the remaining positional features are shown in S7 Fig.

Interestingly, nearly all predictive positional *k*-mers from Fig 5 were also selected as robust and predictive global *k*-mer count features in Fig 3. In particular the poly-U and pyrimidine *k*-mers are among the most predictive *k*-mers for both feature types. However, positional feature plots additionally show that effect strengths of these *k*-mers differ with their position relative to the start AUG. For example, the *U*_1-3_ *k*-mers have an overall positive effect on IRES activity, which is largest if the *k*-mers are located about 50nt upstream of the start AUG.

At the same time, many other features (e.g. CU, UUC, G and CAG) also show positions location-specific effects on IRES activity. Most notably, positional features of these *k*-mers tend to form “islands” from positions at which they have an effect on activity. These islands are consistently located around positions −50 (*k*-mers CU, UC, UCU, CUU, UUC, G, AG and GA) and −150 (*k*-mers G, UA, AG and GA). Interestingly, for the majority of presented *k*-mers, positions with the strongest effect are not located directly upstream of the start AUG. Further, congruence between optimal location for *k*-mers with negative effects (G, AG, GG, GA) and optimal locations for C/U-rich *k*-mers with positive effects further supports our interpretation of the poly-A, purine tract and G/A-rich *k*-mers as anti-correlated with the C/U-rich *k*-mers.

The CAG *k*-mer also shows distinct positional preferences for locations immediately upstream of the start codon. We further investigated its effect to determine whether it is a part of a larger motif, and whether there is a difference in splicing between sequences with and without the CAG *k*-mer. Our analyses (see S1 Text) indicate that the CAG *k*-mer may be related to RNA splicing in the group of dsRNA viruses, but not in Retroviruses.

In addition, a large number of *k*-mers are robust and predictive only for a single sequence group (S7 Fig). Similar to the global *k*-mer features, the unique positional *k*-mers include C-rich *k*-mers C, CC, CUCC, UCC, CUC selected exclusively by the retroviral group. Interestingly, these *k*-mers show positional preferences different from those of the common positional *k*-mers, by forming islands around positions −50 and −200. Finally, we also found that a number of predictive positional *k*-mers are selected uniquely for the group of dsRNA viruses (e.g. AU, ACC, UG, AUU, UAC; S7 Fig); these positional *k*-mers show little consistency in terms of preferred positions, suggesting a different mode of action of IRESs from dsRNA viruses.

## Discussion

**Fig 6:**
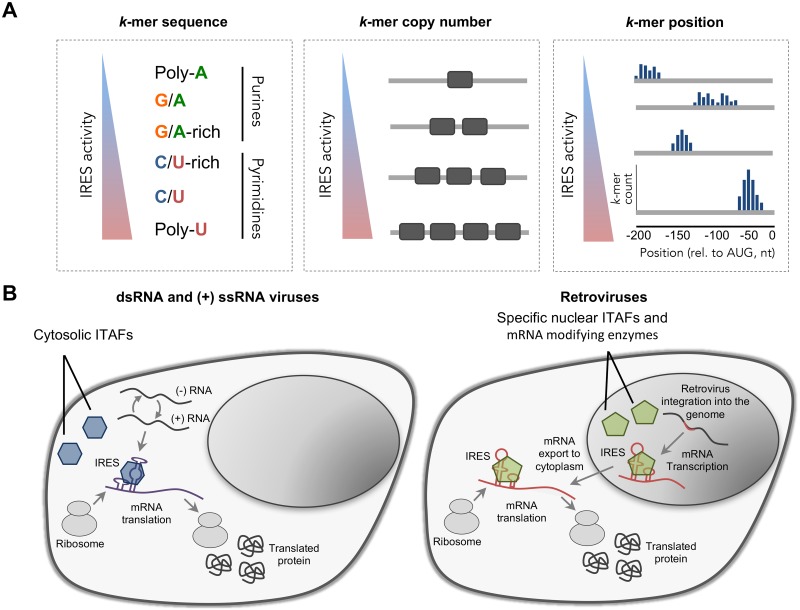
Summary of the sequence features associated with IRES activity. (A) Illustration of the sequence features found by our models and their association with IRES activity: (left) k-mer sequence, (middle) the number of sites of a *k*-mer, and (right) the position of the *k*-mer relative to the AUG start codon. (B) Illustration of the different life cycles of (left) dsRNA/(+) ssRNA viruses and (right) Retroviruses which may have led to differences in their IRESs sequence features. Retroviruses are integrated into the host genome and RNA-PolII transcribes their mRNA in the nucleus. Thus, their IRES elements are exposed to the nuclear environment including mRNA modifying enzymes (methylation, pseudouridylation etc) and nuclear specific ITAFs that can shuttle with the mRNA to the cytoplasm to facilitate cap-independent recruitment of the ribosome. In contrast, dsRNA and (+) ssRNA viruses that spend their entire replication cycle in the cytoplasm are exposed to cytosolic factors, which in turn can facilitate cap-independent recruitment of the ribosome.

In this work we provide the first in-depth computational analysis of thousands of IRESs from the human genome and different types of viruses. Analyses of this largest set of IRESs to date allowed us to decipher the effect of sequence features, their number and position relative to the AUG on IRES activity (summarised in Fig 6A). To achieve this, we trained and interpreted Random Forest models that predict IRES activity from *k*-mer features of RNA sequences.

### Identified *k*-mers resemble ITAF binding motifs

Using the trained models, we identified robust and predictive *k*-mer features, which based on their composition could be divided into two classes: pyrimidine-rich elements, and purine-rich elements (Figs 3A and 6A). Notably, *k*-mers from these classes are generally associated with the same kind of effect on IRES activity: pyrimidine-rich elements tend to have a positive effect on activity, whereas the purine-rich elements tend to have a negative effect.

Interestingly, sequences of predictive pyrimidine-rich *k*-mers resemble consensus binding motifs of known IRES *trans*-acting factors (ITAFs). The poly-U *k*-mers are consistent with the poly-U binding motif described for the hnRNP C1/C2 [57] RNA-binding proteins (RBPs), which were shown to be a part of the protein complex forming the XIAP IRES [58]. Whereas the pyrimidine-rich *k*-mers are consistent with the binding motifs of the PCBP-2 [59], PCBP-1 [60] and PTB-1 RBPs. The PCBP proteins were previously implicated in regulating IRES activity of the hepatitis C virus, poliovirus and rhinovirus IRESs [61], and the human proto-oncogene c-*myc* [62]. And the PTB-1 was previously shown to interact with many cellular and viral IRESs [25], and proposed as an universal ITAF [56]. The correspondence between ITAFs and pyrimidine-rich *k*-mer features, and the strong positive effect of the poly-U and pyrimidine tract *k*-mers on IRES activity (Fig 3A), agree with the proposed role of ITAFs as RNA-binding proteins involved in cap-independent translation initiation.

In accordance with this interpretation, we observed that C/U-rich *k*-mers that contain a single non-C/U nucleotide tend to be associated with increased IRES activity. Given their similarity to the poly-U and pyrimidine tract *k*-mer features, interpreted as potential ITAF binding sites, we propose that the C/U-rich *k*-mer features may represent imperfect binding sites of the PCBP and PTB proteins. This interpretation is supported by the observation that, compared to the perfect C/U-tract *k*-mers, features of this class tend to have a weaker effect on predicted activity.

Notably, systematic measurements of hundreds of fully designed oligos, in which the number of sites of the pyrimidine-rich TEV IRES element was carefully varied, support our finding of the positive relationship between the number of pyrimidine-rich elements and IRES activity. Thus, our study demonstrates the power of combining computational models with systematic measurements of synthetically designed oligos to decipher the principles governing IRES activity.

### IRES architectures differ between virus types

Our results on common and unique sequence features uncover that poly-U and C/U-rich *k*-mers are shared among cellular and viral IRESs, including different families of viruses. This suggests that the involvement of ITAFs these *k*-mers represent in IRES-mediated translation initiation is not limited to a single viral class or location within human transcripts, but is shared across viral classes, as well as between viruses and eukaryotes. However, we also found that for IRESs originating form retroviral genomes, C-rich elements are stronger predictors of high IRES activity than for dsRNA and (+) ssRNA viruses (Fig 3B) and have different positional preferences (S7 Fig).

If pyrimidine tract *k*-mers indeed represent PCBP-1/2 and PTB binding sites, then while binding of these ITAFs to mRNA leads to increased IRES activity irrespective of its virus type, our results suggest that different virus types preferentially rely on different ITAFs for cap-independent translation initiation. The U/C-neutral *k*-mers are more consistent with the *U*[*UC*]*U*[*UC*]_2_ and *C*_2_ *U* PTB binding motifs [56, 63] that have a weaker preference for cytosines, whereas the C-rich *k*-mers are more consistent with the *UC*_3_ *U*_2_ *C*_3_ *U* and *U*_2_ *C*_6_ *AU* PCBP-2 binding motifs [59] showing a stronger cytosine preference. Together this suggests that, compared to other sequence groups, retroviruses preferentially employ PCBP-1/2 RBPs for cap-independent translation initiation.

Interestingly, in contrast to most dsRNA and (+) ssRNA viruses, which spend their entire replication cycle in the cytoplasm, retroviruses are integrated into the host genome and their transcribed mRNA is exposed to the nuclear environment (Fig 6B). Previous reports indicated that some IRESs require a “nuclear experience” in order to be functional [64, 65, 66]. It was suggested that nuclear specific events such as RNA modifications (by methylation, pseudouridylation and others) or the binding of exclusively nuclear ITAFs are required for certain IRESs. Our finding of retroviral IRESs preference for C-rich *k*-mers, presumably recognised by the PCBP ITAF, suggests that the mechanism by which IRES-mediated translation is accomplished, and consequently, IRES architecture, differ between viruses, which were evolved in differed cellular compartments. Taken together with numerous *k*-mer features, which were found to be predictive only for dsRNA IRESs (S6 and S7 Figs), these results provide further support the proposition that viral IRESs arose independently several times in evolution [42]. Since ITAF localisation can be affected both by nuclear membrane disruption and by active nucleo-cytoplasmitic shuttling, further investigation is needed to determine the local concentration of ITAFs and its effect on the evolution of IRES sequence features in different viruses.

### ITAFs exhibit distinct location preferences

When considering positional *k*-mer features, we additionally found that many of the pyrimidine-rich features have a strong positional preference for location islands approximately 50nt and 150nt upstream of the start codon and a similar positive effect on the predicted IRES activity (Figs 5 and 6A). The positive effect of these features, their similarity to ITAF binding motifs, and preference for distinct locations upstream of the start codon collectively suggest that ITAFs, whose (partial) binding motifs these *k*-mers describe, have multiple distinct optimal locations upstream of the start AUG at which they can contribute towards cap-independent translation initiation.

Intriguingly, predictive positions of the C-rich *k*-mers differ from that of the poly-U and U/C-neutral *k*-mers, and show a preference in retroviral IRESs for locations approximately 200nt upstream of the start codon. This further supports our proposition that IRESs originating from retroviral genomes rely more on PCBP-1/2 ITAFs for translation initiation, and suggests their optimal binding location.

### Limitation in detecting RNA structure features as a determinant of IRES activity

In our analyses we were unable to find a strong predictive relationship between RNA secondary structure and IRES activity (see S1 Text), although RNA structure was previously shown to be functionally important for some viral IRESs. There are several possible reasons: First, the high-throughput assay conducted in [21] used designed synthetic oligonucleotides as the input sequence. Thus, the length of the tested sequences was limited to 174nt, which is shorter than some reported long structural viral IRESs [7]. It is possible that the identified IRESs do not form complex secondary structures as reported before (e.g. [67]), therefore limiting our ability to detect structural features in the current dataset. Second, it was shown that IRESs can form dynamic structures and that the binding of ITAFs can induce conformational changes that, in turn, facilitate IRES activity [68]. Thus, *in silico* prediction of RNA structure may differ considerably form the *in vivo* structures in the presence of ITAFs. In addition, computational predictions are limited in the ability to model complex tertiary structures such as pseudoknots. In order to investigate the relationship between RNA structure and IRES activity systematic measurements of secondary structures should be performed on the assayed sequences in cells. Recent advances in technology that facilitate high-throughput structural measurements *in vivo* [69] can shed light on this important layer of IRES regulation.

In this study we demonstrated that RNA sequence is predictive IRES activity, and proposed common and virus type-specific sequence *k*-mer features that may play a functional role in determining IRES activity, and could be used to predict IRESs *in silico*. Our results also yield a high-level IRES architecture of sequence features and their spatial organisation in RNA sequences, which suggests optimal positioning of ITAF binding sites upstream of the start AUG, and may be used to guide future synthetic IRES designs.

## Supporting information

S1 Text Supporting information with extended methods and results. (PDF)

S2 Text The detection of IRESs in Weingarten-Gabbay *et al*. [21]—Controls and supporting evidences from previous studies. (PDF)

S1 Fig IRES activity distribution for all sequences remaining after filtering. Inset plot shows distribution of IRES activity in active sequences (IRES activity above background levels).
(PDF)

S2 Fig Cross-validation scheme employed for training RF models. Rectangular boxes denote actions or procedures, whereas round boxes are used denote their input or output (results); hatched boxes group items that belong to the same CV loop (outer or inner) or CV set (training or testing); arrows show how information flows through the CV procedure, with the arrows crossing CV loop/set boundaries drawn using dashed lines.
(PDF)

S3 Fig Representative examples of partial dependence plots. Three features from the dsRNA viruses models (*k* = 4, averaged over 10 CV folds): features U, AAAA and CAG in [−20, 0] (as shown in the order from left to right) were respectively classified as positive, negative and positive.
(PDF)

S4 Fig Cross-validation performance of *k*-mer count (solid lines) or presence (dashed lines) models trained on human CDS and negative-sense ssRNA viruses sequence groups. (PDF)

S5 Fig Cross-validation performance of models trained on subsamples of sequences from the group of dsRNA viruses. All models use global and positional *k*-mer counts (*k* = 4). Horizontal axis shows the number and the relative percentage of positive IRESs in the dataset, with the leftmost point (106 sequences) corresponding to the relative incidence of positive IRESs in the (−) ssRNA viruses group. Mean performance (solid line) and its standard deviation (shaded area) are shown for 5 random subsamples. These results indicate that small numbers of positive IRESs in a training set can limit predictive power of models trained on that set.
(PDF)

S6 Fig Robust and predictive global *k*-mer features that are uniquely selected by one sequence group. (PDF)

S7 Fig Robust and predictive positional *k*-mer features that are uniquely selected by one sequence group. (PDF)

S8 Fig Expression measurements of 512 designed oligos with increasing copy number of the TEV IRES element. eGFP expression measurements of all the 512 designed oligos with 1-8 copies of the TEV IRES element (A) when placed in a synthetic background and (B) a native background from the human beta-globin (HBB) gene. (C) Joint analysis of the two backgrounds and the two biological replicates. Data was binned into four groups according to TEV sites number and one-way ANOVA was performed to determine if the difference between expression levels of the four bins is significant (*p* < 0.003).
(PDF)

S9 Fig IRES activity across human and viral transcripts. Moving average analysis of the fraction of positive IRESs across the 5′ UTR, coding sequence and the 3′ UTRs of human transcripts and (+) ssRNA viruses encoding a single polyprotein. In contrast to viral transcripts, which present uniform activity level across different regions, different activity level is obtained for human 5′ UTRs, coding sequences and the 3′ UTRs.
(PDF)

S1 Table Sequences of oligos with no IRES elements (i.e. background sequences) used in synthetic designs. (PDF)

S2 Table Annotated dataset of all the synthetic TEV oligos used in the C/U-rich element multiplicity analysis. (TAB)
