# Supplementary material for: Sequence features of viral and human Internal Ribosome Entry Sites predictive of their activity
Source: PLoS Comput Biol. 2017 Sep 18;13(9):e1005734. doi: 10.1371/journal.pcbi.1005734 (PMC5630158; doi:10.1371/journal.pcbi.1005734)
Supplement: S1 Text — (PDF) [file pcbi.1005734.s001.pdf]

Supporting information for "Sequence features of viral and human Internal Ribosome Entry Sites predictive of their activity"

Alexey A. Gritsenko [1,2,3,*], Shira Weingarten-Gabbay [4,5,*], Shani Elias-Kirma [4,5], Ronit Nir [4,5], Dick de Ridder [1,2,3,6,†], Eran Segal [4,5,†]

[1]The Delft Bioinformatics Laboratory, Department of Intelligent Systems, Delft University of Technology, Delft, The Netherlands.
[2]Platform Green Synthetic Biology, Delft, The Netherlands.
[3]Kluyver Centre for Genomics of Industrial Fermentation, Delft, The Netherlands.
[4]Department of Computer Science and Applied Mathematics, Weizmann Institute of Science, Rehovot 76100, Israel.
[5]Department of Molecular Cell Biology, Weizmann Institute of Science, Rehovot 76100, Israel.
[6]Bioinformatics Group, Wageningen University, Wageningen, The Netherlands.

May 13, 2017

1 Data and data pre-processing

In a recent study (Weingarten-Gabbay *et al.*, 2016) we described a high-throughput IRES activity assay that we used to measure IRES activity for thousands of sequences including 28,669 native fragments from the human and viral genomes. Briefly, we obtained a mixed pool of oligonucleotides, 210nt (174nt variable region plus constant primer sequences) in length, using parallel DNA synthesis technology (Cleary *et al.*, 2004; LeProust *et al.*, 2010; Sharon *et al.*, 2012). We then amplified the library using constant primers, cloned it into the lentiviral bicistronic plasmid 12nt upstream of the eGFP (enhanced Green Fluorescent Protein) coding sequence (Fig. 1A) and infected H1299 human lung cells so that each cell integrates a single oligo. In this plasmid mRFP (monomeric Red Fluorescent Protein) is translated in a cap-dependent manner, whereas eGFP translation requires alternative mechanisms. We thus used eGFP expression as a proxy for IRES activity induced by the variable sequence. To obtain eGFP expression we sorted the resulting pool of cells into 16 bins according to eGFP fluorescence, while also filtering based on mRFP fluorescence to control for cell state, and used deep sequencing to compute a score for the expression of each designed oligo based on the distribution of its sequence reads across expression bins. Using this approach, we measured IRES activity of a library of 55,000 sequences.

IRES activity measurements analysed in this work are complemented by high-throughput measurements of splicing activity and promoter activity (Weingarten-Gabbay *et al.*, 2016). As in the original manuscript, these additional measurements were used to filter out unreliable sequences, i.e. sequences whose eGFP expression was likely to be a result of cap-dependent translation due to (i) the mRFP and the assayed sequence being spliced out using a splice acceptor site present in the assayed sequence, or due to (ii) independent transcription of the eGFP from a cryptic promoter in the assayed sequence. To this end, following Weingarten-Gabbay *et al.*, all oligos with splicing scores below -2.5 or promoter activity above 0.2 were removed from the analyses. To further reduce the fraction of oligos, for which eGFP translation could be a result of splicing, we additionally removed all positive sequences (IRES activity above background levels) for which splicing activity could not be measured.

Several filtering and averaging steps were taken in order to obtain more reliable estimates and to increase robustness of the learned sequence models. First, for all analyses measured IRES activities were $\log_2$-transformed and averaged across the two replicates. Then, IRES sequences that had background IRES activity levels in only one of the replicates, and sequences that could be measured in at least one of the replicates were filtered out. Finally, to reduce the affect of outlier sequences with very high IRES activity on the learned predictive models, IRES activities were capped at the 99.5% percentile.

*These authors contributed equally.
†To whom correspondence should be addressed.

Further, because sequences outside of the 174nt variable region can affect IRES activity (e.g. by forming secondary structure with the variable region), for our analyses we extended the variable region by 84nt downstream and 74nt upstream as shown in Fig. 1A (solid filling).

2 Random Forest parameter grid search

When learning random forests, parameters were chosen using a grid search performed on the inner CV loop that evaluated all possible parameter combinations. The learning rate r, minimum number of leaf node training samples m and subsampling fraction f parameters were chosen in this way from grids $[0.001, 0.002, 0.004, 0.008]$, $[5, 25, 125]$ and $[0.9, 0.7]$ respectively.

3 Detailed analysis of the upstream CAG feature

The CAG k-mer in Fig. 5B does not share positional preferences of other features for locations around -50 or -150; instead its effect is strongest when it is located close to the start `AUG` at positions $[-30, 0]$. We expected that if this k-mer is a part of the optimal translation initiation context or splicing signal, then it would show further position or reading frame preferences within the $[-30, 0]$ window. To check this, we analysed CAG position preferences, sequence around CAG, and splicing score difference between sequences with and without CAG for the groups of dsRNA viruses and retroviruses. These groups were chosen as they are the two most specific sequence groups for which this feature was consistently selected across all CV folds and had a strong effect.

First, we compared position distributions for CAG within the $[-20, 0]$ window between positive and negative dsRNA virus sequences. Fig. SI-1A shows a strong preference of the CAG k-mer in dsRNA virus IRES sequences for position -15, i.e. the end of the variable part of the assayed sequences (positions $[-12; 0]$ are the same for all sequences; see Fig. 1A). We then sought to determine whether this k-mer is a part of a larger sequence motif and checked for position-specific nucleotide enrichment between the sets of positive and negative dsRNA virus sequences with a CAG in the $[-20, 0]$ window. Fig. SI-1B shows a significant (Binomial test $p < 0.05$; visualised using the Two Sample Logo website, Vacic *et al.* (2006)) enrichment for Us upstream of the CAG k-mer; the downstream part was not included in the analyses due to the strong preference of the CAG for positions right before the constant part of the sequences. Remarkably, the enriched sequence resembles the canonical splice acceptor motif of poly-U followed by N[CT]AGG (Rosenberg *et al.*, 2015), suggesting that the CAG k-mer may be a part of a splicing site located at the end of analysed IRES sequences.

Presence of such a splicing site may lead to the loss of mRFP and the assayed IRES sequences in spliced mRNAs and result in translation of the eGFP protein through classical cap-dependent initiation mechanisms. To confirm that this is indeed what may be happening, we compared distributions of splicing scores from Weingarten-Gabbay *et al.* (2016), which are indicative of the $\log_2$ splice-in ratios for the assayed sequences, between positive dsRNA virus sequences with a CAG in the $[-20, 0]$ window and without it. Fig. SI-1C shows that IRES sequences with a CAG k-mer in the given window tend to have significantly smaller splicing scores than the sequences without it (Mann-Whitney U-test, $p < 0.001$), suggesting that the +CAG sequences are spliced more often.

We repeated the above analyses for the retroviral group, and found that it only partially recapitulates the results obtained for the group of dsRNA viruses. In particular, while we found a similar poly-U enrichment upstream of the CAG, there was no longer a strong preference for position -15, and the difference in splicing scores between $-$CAG and +CAG sequences was not present. Neither the reason for differences in position preferences between dsRNA viruses and retroviruses, nor a possible mechanism that could link CAG -15 position preference and splicing activity, are clear to us.

Presence of active splicing signals in IRES sequences is problematic for the IRES activity assay, as its measurements may be inflated by eGFP produced via cap-dependent translation mechanisms. However, our analyses of predictive RNA sequence features across different groups of sequences suggest that splicing signals may only moderately effect IRES activity measurements, since only a handful of presented sequence features could be linked to the splicing mechanism. Moreover, the sequence overlap between the splicing acceptor motif and the hnRNAP C1/C2 ITAF binding motifs, both of which require the presence of a poly-U stretch, suggests that co-occurrence of splicing and IRES activity is a general phenomenon. This is supported by the fact that most of the known ITAFs have also been implicated in pre-mRNA splicing (Hernandez, 2008); and by the existence of IRESs, such as XIAP, which are known to contain splice sites (Riley *et al.*, 2010).

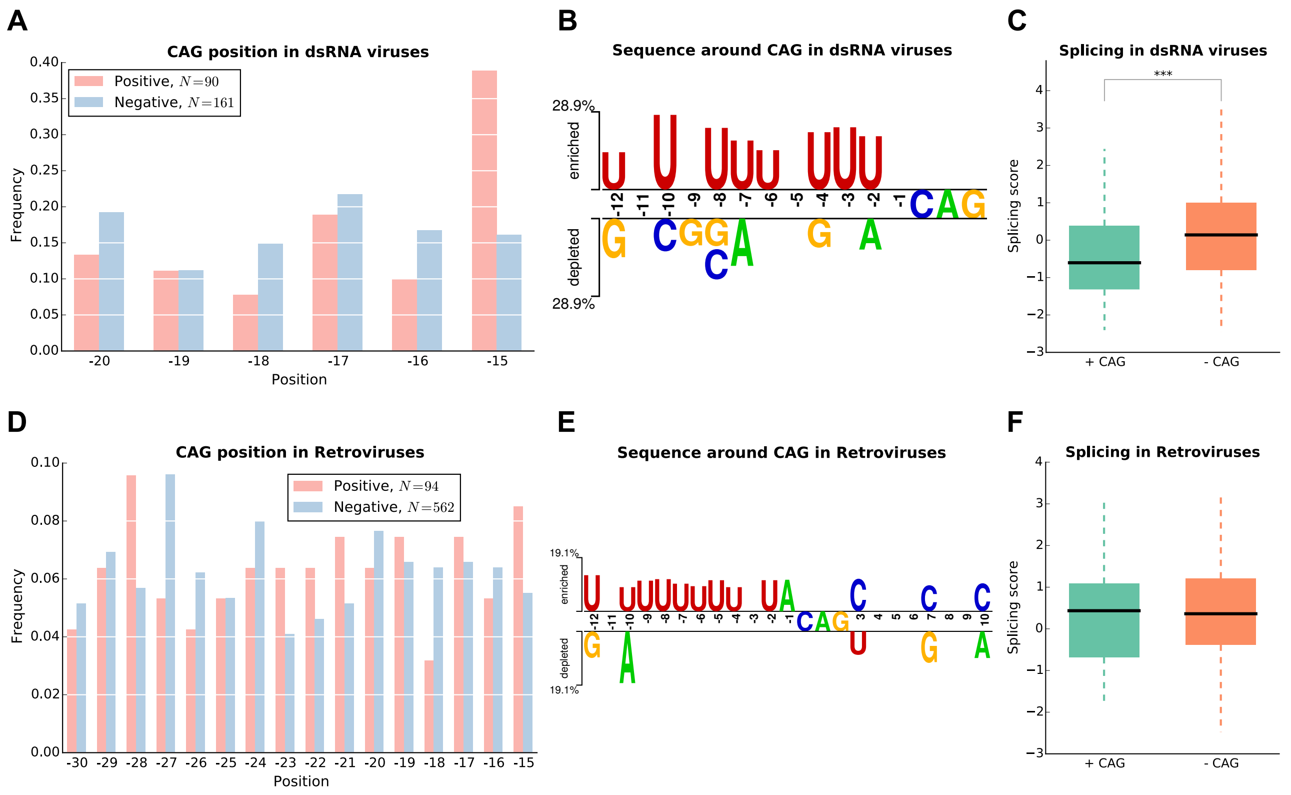

Figure SI-1: Detailed analyses of the CAG k-mer within the $[-20, 0]$ window of the dsRNA viruses group (top row, A-C) and within the $[-30, -10]$ window of the retroviruses group (bottom row, D-F). (A, D) position preference; (B, E) enriched motif around the k-mer; and (C, F) distribution of splicing scores for positive sequences with (+CAG) and without (−CAG) the k-mer in the corresponding window.

4 RNA secondary structure features

Because RNA structure is considered to be a major determinant of IRES activity in known IRESs, we sought to incorporate it in our prediction models. To this end several features describing RNA structure and accessibility were calculated for all sequences and, after applying the same feature pre-selection as in the case of k-mer counts, were used as predictive model features.

4.1 RNA accessibility and region interaction

First, we attempted to describe RNA structures in terms of *accessibility* and *region interactions*. To account for local context effects, secondary structures were predicted for sequences with regions flanking them in the reporter construct (Fig. 1A). RNA base pairing probabilities were computed by folding the *entire* sequence (330nt) using the Vienna RNA package (Lorenz *et al.*, 2011) with default settings.

We defined RNA accessibility of a region as the expected number of unpaired nucleotides in this region. The intuition behind this definition is that if a region is highly paired, it is unavailable for interactions with RNA-binding proteins (RBPs) required to initiate translation or with the ribosome itself. This measure was calculated as region length minus the sum of base pairing probability matrix (BPPM) columns corresponding to that region. Similarly, to capture high-level RNA secondary structure, we defined the RNA interaction measure of two regions as the expected number of paired nucleotides between the two regions; and calculated it as the sum of elements in the BPPM located at the intersection of rows corresponding to the first region and columns corresponding to the second region.

RNA accessibility and interactions features were calculated for 10nt moving windows of the folded sequence. These features showed weak (Spearman $|\rho| \leq 0.13$), but consistent correlations across different sequence groups. Specifically, RNA accessibility shows a reproducible pattern of negative-positive-negative correlation with IRES activity in region $[0, 50]$ and a similar, although weaker, correlation pattern for region $[-270, -230]$ (Fig. SI-2A). Because we expected that RNA accessibility correlations would be easier to interpret as a product of individual region interactions, we also computed correlations between RNA region interaction features and IRES activity (Fig. SI-2B). Correlation pattens observed for region interaction features suggest that (i) pairing between the

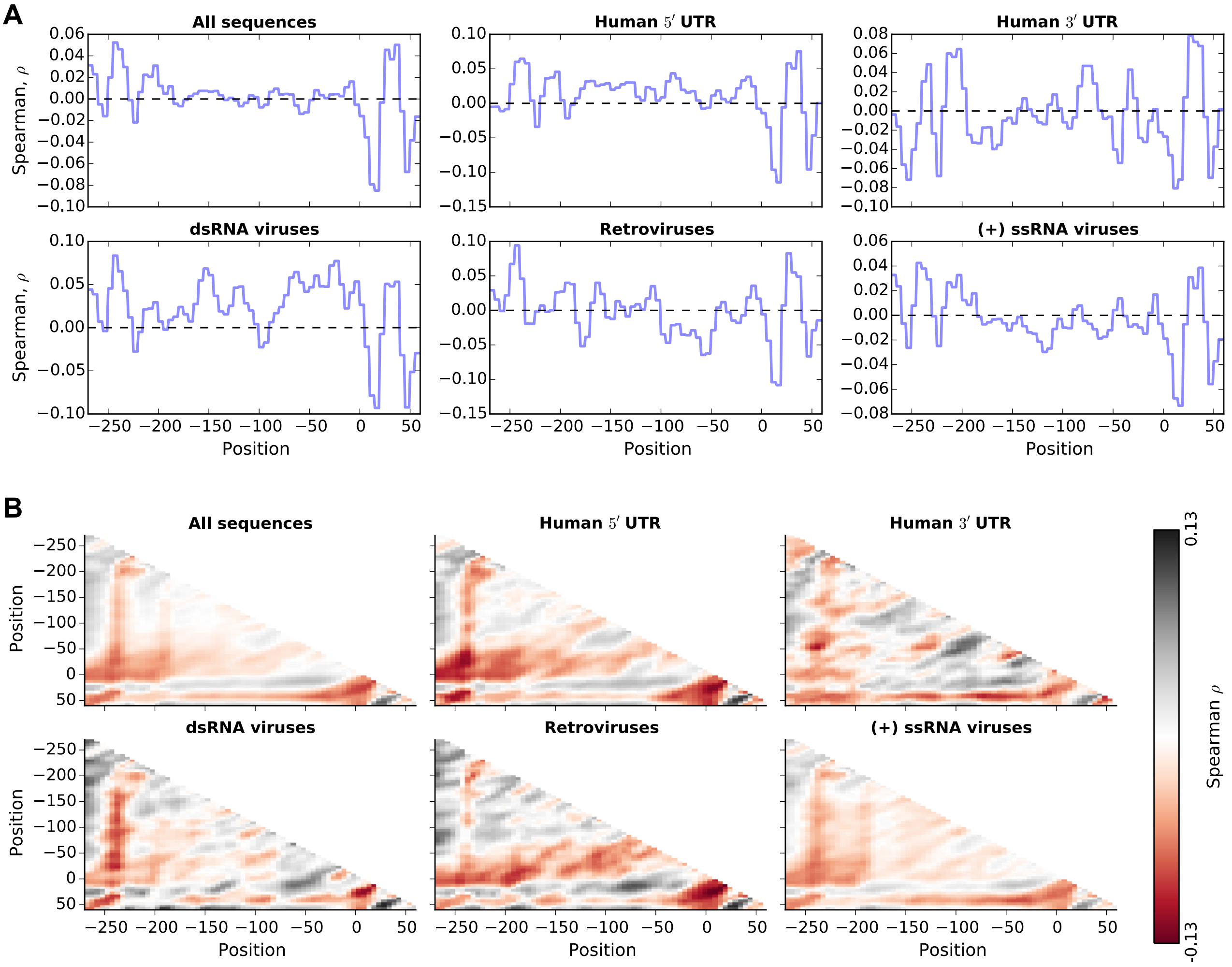

Figure SI-2: (A) RNA accessibility correlations for 10nt moving windows with a step of 5nt; and (B) RNA region interaction correlations for moving 10nt $\times$ 10nt regions with a step of 5nt computed for different sequence groups. Correlations for overlapping windows (regions) were averaged.

region located immediately upstream of position -250 or the region located immediately downstream of position 50 with any other region negatively correlates with IRES activity (predominantly red columns and rows are observed around these positions across all sequence groups); (ii) interactions of regions around the start `AUG` with nearby regions show strongest correlations with IRES activity (as can be readily seen from the dark gray/red spots around the origin for the Retroviruses and Human 5′ UTR sequence groups in Fig. SI-2B). These correlations suggest that the RNA structure formed by the three mentioned regions may play a role in the mechanism of IRES-mediated translation.

Given these observed correlations, we sought to improve our Random Forest models by including RNA accessibility and region interaction features. We followed the same feature pre-selection procedure as described for k-mer features in the main text, and considered different feature combinations, but did not observe any improvement in predictor accuracy beyond what could be achieved using k-mer features alone (see Fig. SI-4A).

4.2 Accessible k-mer counts

Having observed good predictive power of k-mer features and no improvement in predictor performance when naïvely combining RNA structure or accessibility features with k-mer features, we sought to combine the two feature descriptions in a more in a more direct manner. To this end we modified k-mer count features to produce counts of accessible k-mers by summing k-mer accessibilities instead of occurrences. k-mer accessibilities were calculated as RNA accessibility measurements for regions occupied by k-mer occurrences and normalised by k-mer length. To include accessible k-mer count features in our models, we followed the same feature pre-selection and combination procedure as described for k-mer count features. Unfortunately, as in the case of RNA accessibility and interaction features, we did not observe an increase of model predictive power beyond

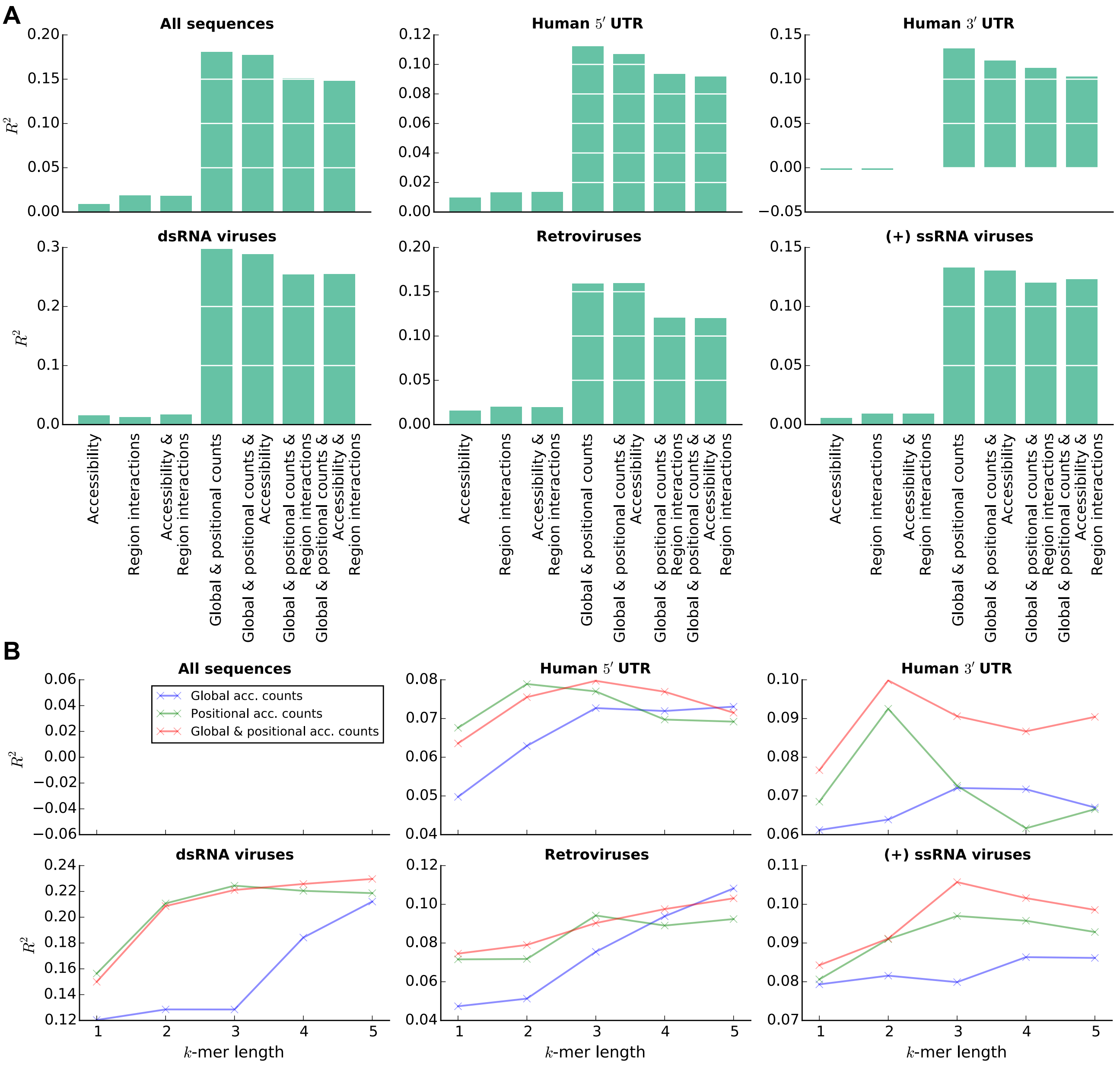

Figure SI-3: Performance of RF predictors trained with RNA structure features. Cross-validation performance of models for difference groups of sequences trained on combinations of (A) k-mer count for $k = 4$, accessibility and region interaction features; and (B) accessible k-mer count features.

what can be achieved by k-mer count features alone (see Fig. SI-4B).

5 Group sequence permutation

Separation of sequences into $n = 7$ groups based on their species and origin resulted in differences in predictive power between groups. These differences may arise due to group-specific IRES mechanisms being captured by the learned models, or due to group structure (i.e. the number of positive and negative sequences). To see whether the observed variation in the defined groups is higher than the variation one would expect from group structure alone, we performed 10 permutation experiments. In each experiment positive and negative sequences were independently permuted across groups, thus preserving group structure, and models were learned on the permuted groups for each combination of features and k-mer lengths as before. Cross-validation (CV) predictive power of models learned on the permuted groups were used to obtain 10 samples of variation that can be expected due to group structure alone (green boxplots in Fig. SI-4). These samples were used to arrive at p-values for the variation observed in defined groups (orange dots in Fig. SI-4) by assuming that they follow a scaled χ^2 distribution with $n - 1$ degrees of freedom and scaling factor $\frac{n-1}{\sigma^2}$, where σ^2 is the unknown true variance

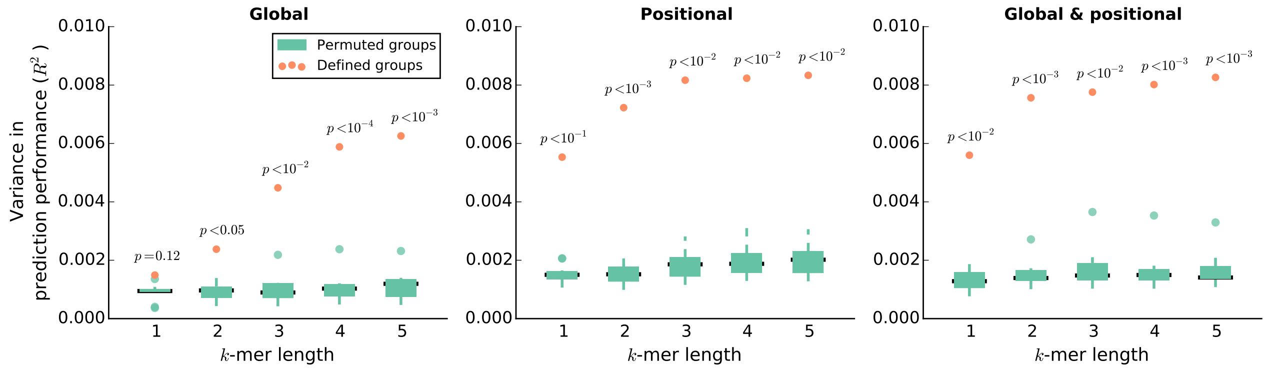

Figure SI-4: Variation in predictive power (R^2) between groups for randomly permuted (green) and defined (orange) groups for different feature combinations (left, middle and right sub-figures) and k-mer lengths. Observed variations in the defined groups are annotated with the corresponding p-values.

estimated as mean of the 10 permutation variances (Knight, 1999). Fig. SI-4 shows that variation observed in defined groups is significantly higher than what can be expected due to group structure alone for the majority of feature and k-mer length combinations ($p < 0.05$ for $k > 2$), suggesting that the sequence groups we defined in the main text capture group-specific mechanisms of IRES translation.

6 Feature importance and partial dependence

When training a RF, tree node variables and splits are chosen to maximise the reduction in weighted variance between the node itself and the two children produced by the node split. Formally, if p, l and r are respectively the current node, and its left and right children; and $S^n_{v,s} = \{(x, y)\}$ are the sets of training samples assigned to nodes $n = p, l, r$ created for feature v and split s, and given as (feature vector, IRES activity) pairs, then feature v and split s are chosen for node p (concisely written as $V(p) = v$ and $S(p) = s$) by maximising

$$C^p = \mathrm{Var}_{(x,y)\in S^p}(y) \cdot |S^p| - \left[\mathrm{Var}_{(x,y)\in S^l}(y) \cdot \left|S^l\right| + \mathrm{Var}_{(x,y)\in S^r}(y) \cdot |S^r|\right],$$

where $\mathrm{Var}_{(x,y)\in S^n}(y)$ gives the variance off all IRES activity values in S^n, and $|S^n|$ gives the number of elements in S^n. Intuitively, the more a variable v is used in the RF trees, and the higher the values C^p are for nodes associated with this variable, the more predictive of IRES activity it is. For our analysis we used *feature importance* as defined in Hastie *et al.* (2005), which captures this intuition by accumulating values C^p for all RF trees $t \in T$ and all nodes p assigned to variable v when calculating its importance I_v:

$$I_v = \frac{1}{|T|} \sum_{t\in T} \frac{\sum_{p\in\{p|p\in t \wedge V(p)=v\}} C^p}{\sum_u \left[\sum_{p\in\{p|p\in t\wedge V(p)=u\}} C^p\right]}.$$

These feature importances were additionally normalised by the maximum I_v to allow for comparison of feature importances between models trained on different sequence groups:

$$\tilde{I}_v = \frac{I_v}{\max_u I_u}.$$

A Random Forest $f(x) = f\left(\left[x^1 \dots x^M\right]\right)$ trained on samples $\{(x_j, y_j)|j = 1 \dots N\}$ and M features can be used to investigate the relationship between each its features and the RF prediction. In order to understand the relationship between the ith variable and the prediction $f(x)$ we considered its *partial dependence* on the RF prediction function f, as described in Hastie *et al.* (2005):

$$f_i\left(x^i\right) = \mathbb{E}_{x^i}\left[f\left(x^1, \dots, x^i, \dots, x^M\right)\right],$$

which for RFs can be efficiently estimated using the training samples x_j as

$$\hat{f}_i\left(x^i\right) = \frac{1}{N}\sum_{j=1}^{N}\left[f\left(x^1_j, \dots, x^i, \dots, x^M_j\right)\right].$$

We used the latter estimation in our model interpretation analyses.

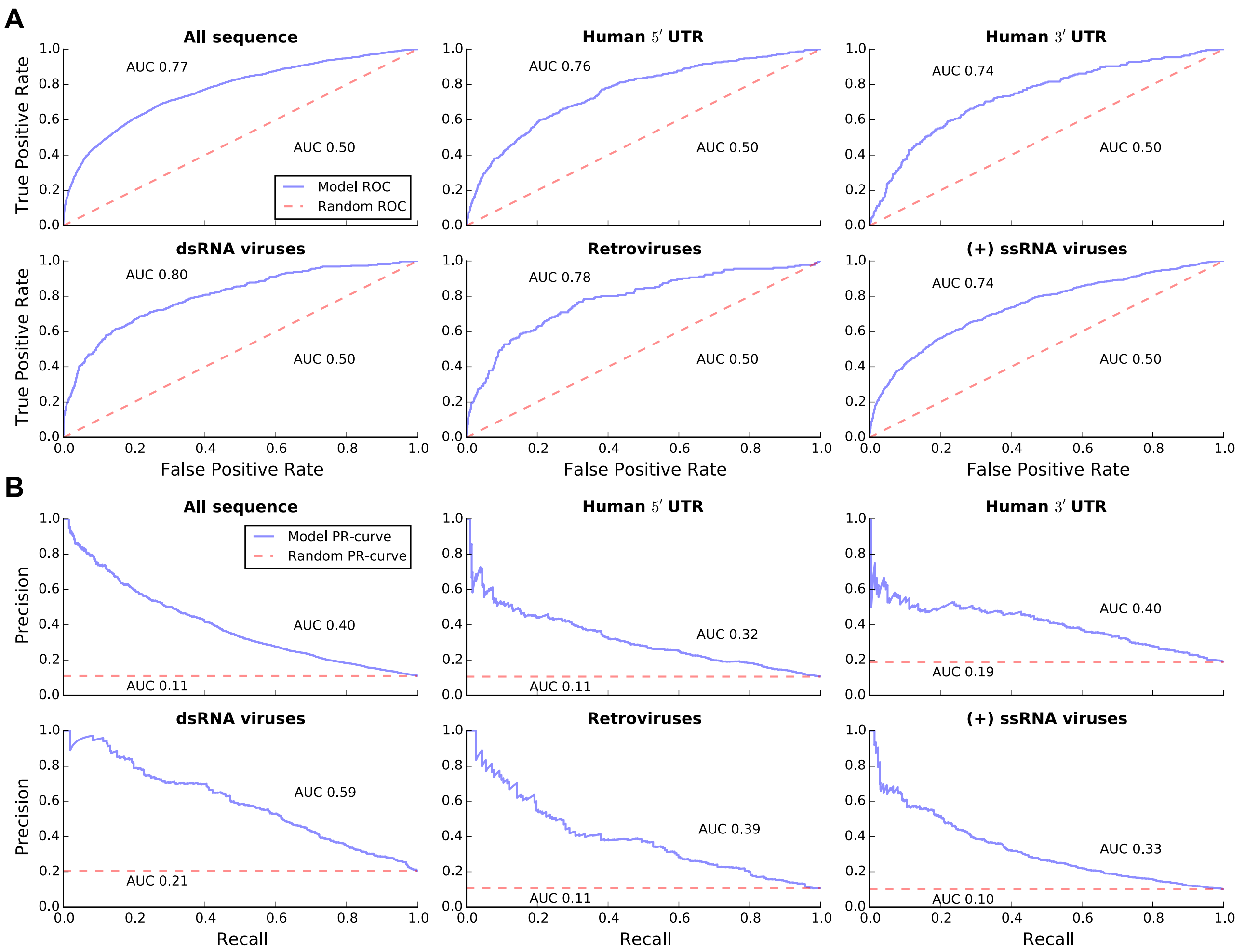

Figure SI-5: Cross-validation performance of the trained RF models selected for interpretation in the main text, as evaluated by (A) the Receiver Operating Characteristic (ROC) curve, and (B) the Precision-Recall (PR) curve. Curves for the trained models are shown as blue lines, and are compared to curves for random predictions (red dashed lines).

7 Model evaluation using the AUC-ROC and AUC-PR measures

To assess the models ability to separate positive and negative IRESs (i.e. sequences with IRES activity above and below background levels) in addition to their ability to predict exact IRES activity levels (as assessed by the R^2, Pearson r and Spearman ρ correlation measures), we calculated the Area Under the Receiver Operating Characteristic curve (AUC-ROC) and the Area Under the Precision-Recall curve (AUC-PR) metrics for our models. These metrics are commonly used to evaluate performance of binary classifiers, with the latter typically employed when the classification problem is imbalanced (Davis and Goadrich, 2006), i.e. has many more samples in one class than in the other. The use for AUC-PR is particularly meaningful for the data available for training our models, as it contains many more negative IRESs than the positive ones ($\approx 89\%$ of measured sequences have background activity levels).

Because the AUC-ROC and AUC-PR measures are not directly applicable to the evaluation of regression models, such as the Random Forest regression models used in the manuscript, we use the models predicted IRES activity to assign a sequence to the positive or negative class based on a decision threshold. Iterating over all possible thresholds then allowed us to construct the ROC and PR curves, as shown in Fig. SI-5 (blue lines). Area under these curves then gives the sought AUC-ROC and AUC-PR measures. A similar procedure was used to determine the ROC and PR curves of random predictions. As can be seen from Fig. SI-5, our models perform significantly better than random predictions, indicating that they generalise well on unseen data.

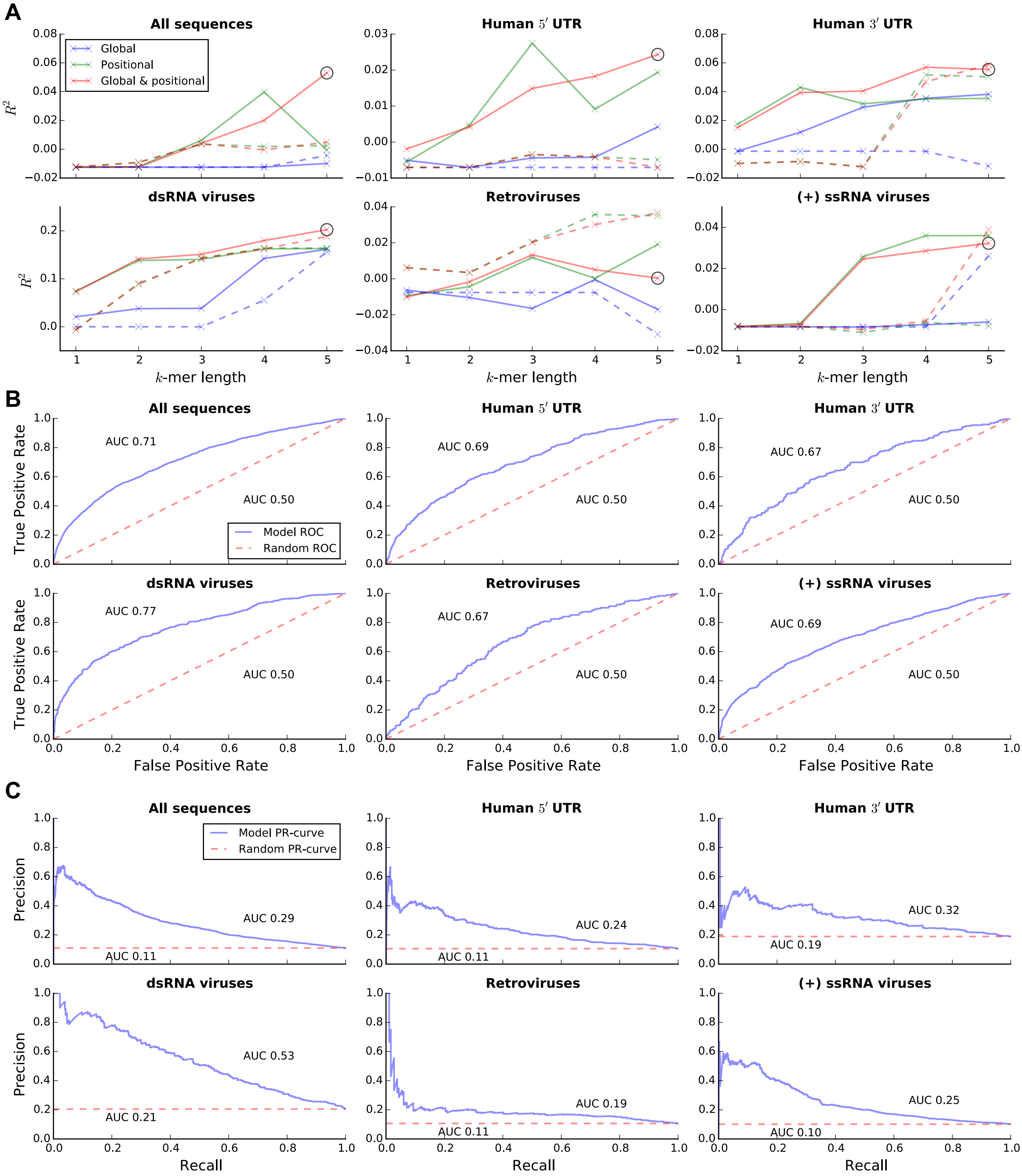

Figure SI-6: Cross-validation performance of SVR models. (A) For different groups of sequences, feature combinations and k-mer lengths. Selected combinations are marked with a circle. k-mer count features are depicted as solid lines, whereas k-mer presence features are depicted as dashed lines. (A) the Receiver Operating Characteristic (ROC) curve and (B) the Precision-Recall (PR) curve for the selected combinations. Curves for the trained models are shown as blue lines, and are compared to curves for random predictions (red dashed lines).

8 Comparison to Support Vector Regression

We sought to compare the RF regression approach, employed for modelling IRES activity and identifying sequence features predictive of it, to alternative machine learning methods. We chose to compare RF regression to Support Vector Regression (SVR) (Drucker *et al.*, 1997), as it is an established regression method that allows for

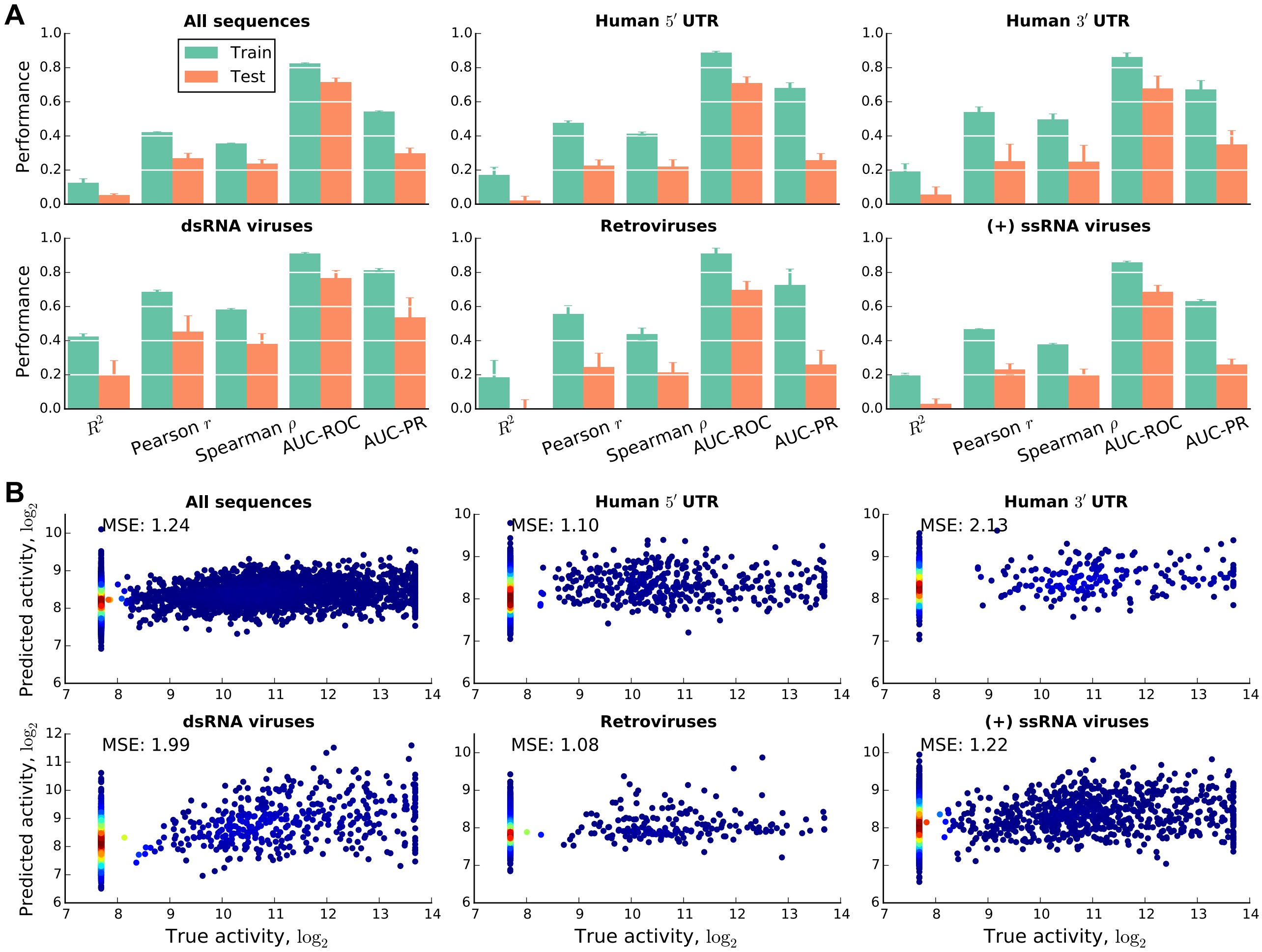

Figure SI-7: (A) Training and test performance of selected SVR models for each of the sequence groups. (B) Scatter plots of true vs. predicted IRES activity for these models with colour representing the local point density (blue to red as low to high density). Mean Squared Error (MSE) for each of the scatter plots is also provided.

construction of interpretable models, thus providing a fair comparison to RF. To ensure that the the evaluation is as fair as possible, we kept all the steps performed prior to model training (i.e. data pre-processing, filtering and k-mer feature pre-selection) the same, and only swapped the RF model training by that of ϵ-SVR (Drucker *et al.*, 1997). Further, the models were trained on the same sequences groups, feature combinations and for the same k-mer lengths as the RF models in the main text.

Optimal parameters for the SVR models were determined using grid search maximising the R^2 metric inside the double-loop CV described before. Model parameters ϵ and C were sought among the following values: $\epsilon \in \{0.001, 0.003, 0.009, 0.027, 0.081, 0.243\}$ and $C \in \{0.027, 0.081, 0.243, 0.729\}$. This range of values allowed for achieving some of the best results observed for SVR on the considered problems (data not shown) at reasonable computational cost. To keep the resulting models interpretable, we used a linear version of the SVR (i.e. no kernels employed). Finally, prior to training, all features and the $\log_2$ IRES activities we rescaled to the range $[-1, +1]$; feature scaling based on the training set was also applied at model evaluation time, and the predictions were scaled back to their original value range.

Performance of the trained models is shown in Fig. SI-6. It can be seen from the figure that, compared to the RF models, SVR achieves inferior performance in terms of the R^2, and the AUC-ROC and AUC-PR metrics demonstrated for models with global and positional 5-mer features. We note that unlike the RF models, the performance of SVR does not level off at $k = 5$ and could possibly improve with longer k-mers. However, since the fraction of sequences with an exact match for k-mer of this length approaches 10^{-3} (see Fig. SI-10), it is unlikely that further performance improvement could be achieved with long k-mer without changing the feature representation (e.g. by allowing inexact k-mer matches). Together, the results of the comparison suggest that for the available data, and the k-mer feature representation described in the manuscript, unless model interpretability can be sacrificed by using SVR kernels, Random Forests should be preferred over SVR for modelling IRES activity.

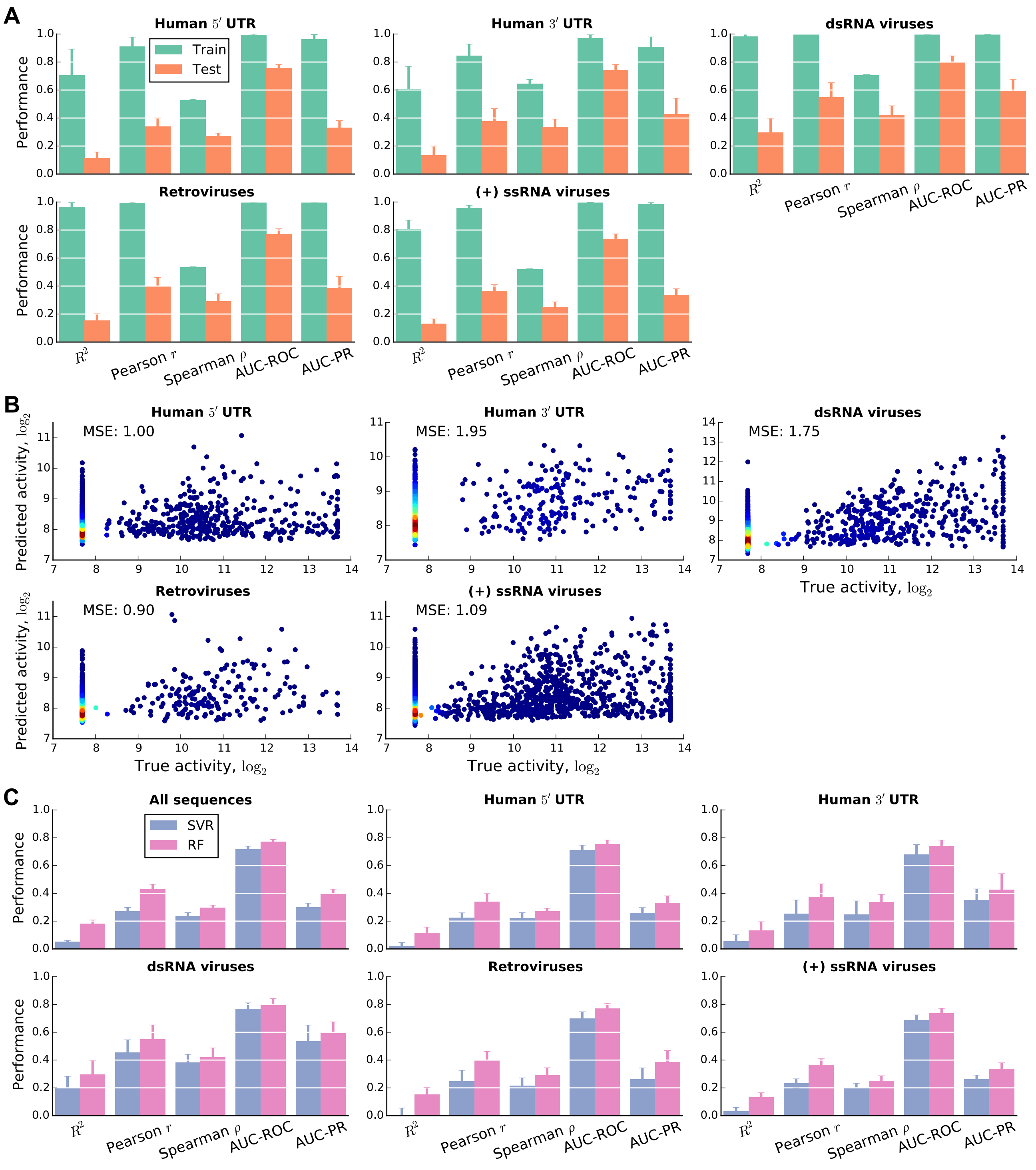

Figure SI-8: (A) Training and test performance of RF models selected for interpretation in the main text for each of the sequence groups. (B) Scatter plots of the true vs. predicted IRES activity for these models with colour representing the local point density (blue to red as low to high density). Mean Squared Error (MSE) for each of the scatter plots is also provided. (C) Comparison of the SVR and RF model performance for all sequence groups and metrics.

For completeness, we also present the scatter plots of the predicted and measured IRES activity, and a summary figure with all considered evaluation metrics for the chosen SVR models in Fig. SI-7. We also provide the corresponding plots for the RF models described in the main text (Figs. SI-8A and B), and finally, to facilitate comparison, we include a visualisation of the cross-validation performance for both modelling approaches in Fig. SI-8C.

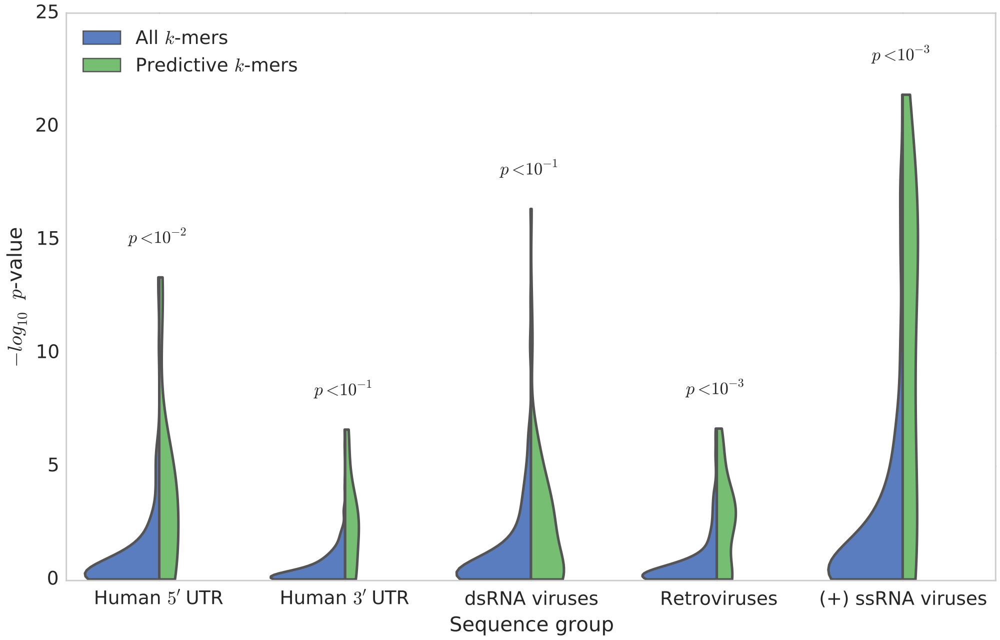

Figure SI-9: Distribution of the 4-mer enrichment p-values for all possible k-mers (blue) and k-mers identified as predictive by the corresponding models. Numbers above the violin plots indicate the one-sided Mann-Whitney U-test p-values for the difference between the two distributions.

9 Comparison of the predictive and enriched k-mers

Using the Random Forest training approach (see Fig. 1 and Material and Methods section of the manuscript) we identified robust and predictive k-mer features for each of the considered sequence groups. These k-mer features were then interpreted in terms of their effect on IRES activity suggested by the trained models (see Results and Discussion sections of the manuscript). Although these features were selected based on a modelling procedure that learns to predict IRES activity from sequence, and through this should be related to IRES mechanisms, we sought to obtain further confirmation of this. To this end we calculated p-values for the enrichment of k-mers (present anywhere in the sequence) in the set of positive sequences (IRES activity above background levels) relative to the set of negative sequences (background IRES activity) for all possible 4-mers using the Fisher's exact test, and compared the distribution of these p-values between all possible 4-mers and the subset of 4-mers identified as robust and predictive by our models. As expected (see Fig. SI-9), on average, the 4-mers identified by the RF models tend do have more significant p-values than the set of all possible 4-mers (all differences between the p-value distributions are significant according to the Mann-Whitney U-test), further emphasing that the k-mers uncovered by our models do not stem from high abundance in the analysed dataset, but are true predictive features of IRES activity.

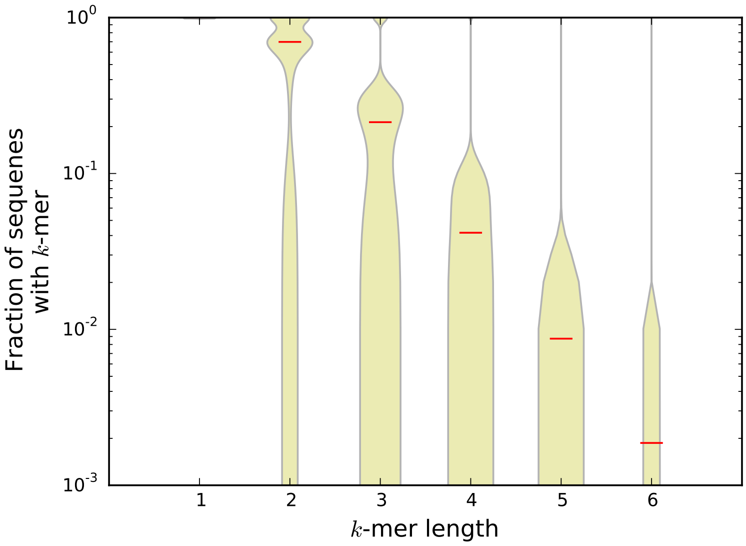

Figure SI-10: Distribution of the fraction of all measured sequences with a specific k-mer present in a specific positional sequence window, shown for all possible k-mers ($k \leq 6$) and all windows. Separate distributions are shown as violin plots for each k-mer length; and red horizontal bars indicate the median fraction for the given length.

References

Cleary, M. A., Kilian, K., Wang, Y., Bradshaw, J., Cavet, G., Ge, W., Kulkarni, A., Paddison, P. J., Chang, K., Sheth, N., *et al.* (2004). Production of complex nucleic acid libraries using highly parallel *in situ* oligonucleotide synthesis. *Nature Methods*, **1**(3), 241–248.

Davis, J. and Goadrich, M. (2006). The relationship between Precision-Recall and ROC curves. In *Proceedings of the 23rd international conference on Machine learning*, pages 233–240. ACM.

Drucker, H., Burges, C. J., Kaufman, L., Smola, A. J., and Vapnik, V. (1997). Support vector regression machines. In *Advances in neural information processing systems*, pages 155–161.

Hastie, T., Tibshirani, R., Friedman, J., and Franklin, J. (2005). The elements of statistical learning: data mining, inference and prediction. *The Mathematical Intelligencer*, **27**(2), 83–85.

Hernandez, G. (2008). Was the initiation of translation in early eukaryotes IRES-driven? *Trends in biochemical sciences*, **33**(2), 58–64.

Knight, K. (1999). *Mathematical Statistics*. Chapman & Hall/CRC Texts in Statistical Science. Chapman and Hall/CRC, 1 edition.

LeProust, E. M., Peck, B. J., Spirin, K., McCuen, H. B., Moore, B., Namsaraev, E., and Caruthers, M. H. (2010). Synthesis of high-quality libraries of long (150mer) oligonucleotides by a novel depurination controlled process. *Nucleic acids research*, **38**(8), 2522–2540.

Lorenz, R., Bernhart, S. H., Zu Siederdissen, C. H., Tafer, H., Flamm, C., Stadler, P. F., Hofacker, I. L., *et al.* (2011). ViennaRNA Package 2.0. *Algorithms for Molecular Biology*, **6**(1), 26.

Riley, A., Jordan, L. E., and Holcik, M. (2010). Distinct 5′ UTRs regulate XIAP expression under normal growth conditions and during cellular stress. *Nucleic acids research*, **38**(14), 4665–4674.

Rosenberg, A. B., Patwardhan, R. P., Shendure, J., and Seelig, G. (2015). Learning the Sequence Determinants of Alternative Splicing from Millions of Random Sequences. *Cell*, **163**(3), 698–711.

Sharon, E., Kalma, Y., Sharp, A., Raveh-Sadka, T., Levo, M., Zeevi, D., Keren, L., Yakhini, Z., Weinberger, A., and Segal, E. (2012). Inferring gene regulatory logic from high-throughput measurements of thousands of systematically designed promoters. *Nature biotechnology*, **30**(6), 521–530.

Vacic, V., Iakoucheva, L. M., and Radivojac, P. (2006). Two Sample Logo: a graphical representation of the differences between two sets of sequence alignments. *Bioinformatics*, **22**(12), 1536–1537.

Weingarten-Gabbay, S., Elias-Kirma, S., Nir, R., Gritsenko, A. A., Stern-Ginossar, N., Yakhini, Z., Weinberger, A., and Segal, E. (2016). Systematic discovery of cap-independent translation sequences in human and viral genomes. *Science*, **351**(6270), aad4939.
