# Supplementary material for: Sequence features of viral and human Internal Ribosome Entry Sites predictive of their activity
Source: PLoS Comput Biol. 2017 Sep 18;13(9):e1005734. doi: 10.1371/journal.pcbi.1005734 (PMC5630158; doi:10.1371/journal.pcbi.1005734)
Supplement: S2 Text — (PDF) [file pcbi.1005734.s002.pdf]

The detection of IRESs in Weingarten-Gabbay *et al.* – controls and supporting evidences from previous studies

The study that generated the data used for the analyses in this manuscript (Weingarten-Gabbay *et al.* Science 2016 (1)) employed a high-throughput bicistronic reporter assay for the detection of multiple IRES sequences. Using bicistronic reporter DNA constructs should be done cautiously and with appropriate controls to avoid false positive results stemming from artifacts of cryptic promoters and splicing sites. Here, we elaborate on the thorough controls conducted to exclude these artifacts and provide multiple examples of high agreement between our findings and other studies in the field.

The main points discussed are:

- Controls for aberrant eGFP expression stemming from cryptic promoter or splicing activity.
- Consistency between the regulatory regions identified in our study and previous studies of five characterized IRESs: ODC1, RUNX1, hSNM1, AEV and Cat-1.
- Consistency between IRES activity of EMCV fragments and the reported activity of the GNRA tetraloop.
- Previously reported viral IRESs in the length of ~200nt.

Unless stated otherwise, the figures discussed below refer to Weingarten-Gabbay *et al.* (*1*)

Controls for aberrant eGFP expression stemming from cryptic promoter or splicing

A large part of our study was dedicated to extensive controls to detect potential artifacts of cryptic promoter and splicing activity, including: (a) high-throughput assay for promoter activity in which the entire library (55,000) was cloned into a bicistronic plasmid lacking an endogenous promoter (Fig 1); (b) high-throughput assay for splicing activity in which reduction in the intact bicistronic mRNA transcript was quantitatively measured using deep sequencing of the cDNA and gDNA (Supplementary Fig S3); (c) qRT-PCR on the entire eGFP(+) population with three different sets of primers probing the mRFP cistron in various locations (Supplementary Fig S4); (d) qRT-PCR on isolated clones expressing individual IRESs with primers on the eGFP and the mRFP cistrons (part of Supplementary Figs S8 and S12); (e) validation of selected IRESs in a bi-cistronic luciferase reporter plasmid (Supplementary Figs S2, S13 and S15); and (f) validation of selected IRESs in a mono-cistronic luciferase plasmid (Supplementary Fig S13).

The following findings demonstrate that the majority of the eGFP signal in our study does not stem from aberrant cap-dependent translation:

(1) **The obtained distribution of cDNA/gDNA ratios of positive eGFP variants.** In our study, we devised a high-throughput assay to identify cryptic splicing events by quantifying the reduction in the levels of intact bicistronic transcripts in cells using constant primers flanking the designed oligo (Supplementary Fig S3a). For variants with no splicing activity we would expect similar levels of cDNA and gDNA reads and thus their ratios should be distributed normally with a mean of 1 (0 in $\log_2$ space) and a standard deviation (std) that represents the biological and technical noise of the assay. Since our primers capture only intact bicistronic mRNAs, variants that undergo

splicing should yield lower numbers of cDNA reads than gDNA reads resulting in lower cDNA/gDNA ratios. Examining the cDNA/gDNA ratios (Supplementary Fig S3b) reveals a normal distribution with mean=1 and a non-symmetric left tail as expected for a population of sequences for which a small fraction of the variants have splicing activity.

It should be noted that, as in any other assay, the output measurements do not match one exact value but form a normal distribution with this value as the mean, i.e. we cannot expect that all the non-spliced variants will get a perfect ratio of 1. Thus, our aim was not to assign an exact cDNA/gDNA ratio for each variant, but rather to determine the probability that its score follows the normal distribution of non-spliced variants. To this end, we fitted a normal distribution using the right side, which represents the variation stemming from the biological and technical noise only. Although many studies use a distance of 2 standard deviations (stds) as the cut-off to determine if a data point originates from a sample's normal distribution, we chose a stricter cut-off and excluded sequences with distance greater than 1.5 stds. Notably, our assay successfully captures XIAP and eIF4G1, for which splicing activity was reported before.

(2) **The distribution of cDNA/gDNA ratios of designed sequences from [+]ssRNA viruses.** Since [+]ssRNA viruses spend the entire life cycle in the cytoplasm, their genome has not evolved specifically to recruit RNA Pol II or the splicing machinery. In addition, there is a strong consensus in the field about the existence of viral IRES-elements. Thus, we tested the similarity between the [+]ssRNA sequence distribution and the fitted normal distribution used to determine non-spliced variants in the library (Supplementary Fig S3b). We found no significant difference ($p>0.5$), providing additional evidence that scores in that range do not represent true splicing.

(3) **A lack of correlation between cDNA/gDNA ratio and eGFP expression.** The strength of the 3' splice-site is a continuous variable ranging from very weak sites, yielding only a few percent of undetectable capped monocistronic mRNA, to strong sites, in which most of the mRNA is being spliced, such as the case of XIAP in our bicistronic reporter (Supplementary Fig S4). If indeed eGFP expression was a result of spliced monocistronic mRNA, we would expect a correlation between the cDNA/gDNA ratio, which represents the 3' splice-site strength, and eGFP expression. Investigating the relationship between these two measurements we found no correlation ($R = -0.07$), demonstrating that for most of the tested oligos eGFP expression does not stem from cryptic splicing (Supplementary Fig S3c).

(4) **qRT-PCR measurements with three sets of primers on the mRFP cistron**. We show that the mRNA levels of the mRFP cistron in eGFP(+) library cells are similar to those of cells infected with the empty vector, which does not drive the expression of eGFP. To ensure that our measurements successfully capture splicing events we tested cells expressing the XIAP IRES, which harbors a cryptic splice site, and obtained clear substantial reduction in the mRNA levels of mRFP. To account for the amount of integrated construct in cells we performed qRT-PCR on the gDNA and found similar levels of the integrated reporter cassette in all the tested samples. This result demonstrates that the majority of the putative IRESs composing the eGFP(+) population do not contain cryptic splice sites (Supplementary Fig S4).

(5) **qRT-PCR on isolated clones expressing individual library oligos with primers on the eGFP and the mRFP cistrons.** We compared the mRNA levels of the first and second cistrons using qRT-PCR on cells expressing 7 individual IRESs from the library. To this end we designed primers that anneal at the end of the first cistron (mRFP) and the beginning of the second cistron (eGFP). As negative and positive controls for splicing we used cells expressing the empty vector or the XIAP IRES, respectively. We found that for 6 of the 7 oligos tested the ratio between the eGFP and the mRFP cistrons is ~1, as expected for cells that do not express monocistronic transcripts and as obtained for the empty vector (Supplementary Fig S8c and Fig S12).

Consistency between the regulatory regions identified in our study and previous studies of characterized IRESs

In our study, we identify the regulatory elements of known and novel IRESs using systematic scanning mutagenesis, each time mutating a window of 14nt so that each nucleotide in this region is mutated randomly to one of the other three nucleotides. To assess the accuracy in detecting functional sequences we carefully investigate the literature for five previously reported IRESs (Figs 3b and 3c). We found that the regulatory regions identified in our study agree with the current knowledge as summarized below:

(1) **The ODC1 IRES** was studied by Pyronnet *et al.* (Mol Cell. 2000(2)). The authors found that mutating two 'UUUC' elements into 'AAAC' inhibited translation from this IRES. In our assay, the two mutated windows that resulted in the lowest expression contained these two 'UUUC' elements, thus consistent with the finding by Nahum Sonenberg's group (Fig 3b).

(2) **The RUNX1 IRES** was studied by Pozner *et al.* (Mol Cell Biol. 2000 (*3*)). Although the authors did not dissect the location of the functional IRES within the ~1,500nt RUNX1 5'UTR, they hypothesized that the functional region resides at the 3' end of the 5'UTR. They report on three "IRES characteristic motifs" in this region: (a) oligopyrimidine tract, (b) a short sequence, 'UUUCC', which is complementary to the 3' end of the 18S rRNA, and (c) a Y-shaped stem-loop structure upstream of the start codon. Thus, our results are in line with the authors hypothesis and provide the first experimental evidence that the 3' end of the RUNX1 5'UTR indeed harbors a functional regulatory region (Fig 3c).

(3) **The hSNM1 IRES** was studied by Zhang *et al.* (DNA repair. 2002 (*4*)). The authors show that the regulatory elements reside somewhere between nucleotides 789-918 of the 5'UTR as deleting this region leads to reduction in IRES activity. Thus, our findings of regulatory region in positions 804-817 are in line with this report (Fig 3c).

(4) **The AEV IRES** was studied by Bakhshesh *et al.* (J Virol. 2008 (*5*)). The authors have shown experimentally that the functional AEV IRES lies within nucleotides 100-494 of the 5'UTR. In addition, they provide evidence for the formation of an HCV-like secondary structure through mutational analysis. Thus, our findings of an active IRES sequence within nucleotides 147-320 and its classification as a "structural IRES" based on scanning mutagenesis analysis are consistent with the current knowledge (Fig 3c).

(5) **The Cat-1 IRES** was studied by Majumder *et al.* (Mol Cell Biol. 2009 (*6*)). The authors found that its function depends on a shift of the IRES into an active structural conformation, which involves the binding of PTB and hnRNP L complex. Thus, our scanning mutagenesis-based classification of the Cat-1 IRES as structured sequence is in line with these findings. In addition, the authors also characterize the PTB binding site and demonstrate that mutating its sequence reduces IRES activity. In line with their findings, we also obtain reduction in expression when mutating this site (positions 61-66 of the tested oligo) (Fig 3c).

Obtaining IRES activity for EMCV IRES fragments containing the GNRA tetraloop

Our measurements of 174nt-long fragments from the EMCV IRES indicate a functional region in positions 151-424 of the reported IRES (Supplementary Fig S6a). A concern was raised about this finding, suggesting that the EMCV fragments harbor cryptic splice sites. Interestingly, the detected region contains the "GNRA tetraloop" that was found to be essential for the EMCV IRES in an extensive study that generated and assayed hundreds of constructs in which the wild-type GNRA sequence (GCGA) was mutated to 'NNNN' (Robertson *et al.* RNA. 1999 (*7*)). Remarkably, this study found that even point mutations in the GNRA tetraloop completely abolished the activity of the EMCV IRES. Since Robertson *et al.* assayed the activity of the wild-type and mutated constracts both *in-vivo* and <u>*in-vitro*</u> using T7 *in-vitro* transcription/translation (TNT) system (Figure 5 of Robertson *et al.* RNA. 1999 (*7*)), the detected GNRA-dependent activity of the EMCV IRES could not be a result of cryptic promoter or splice site. Thus, our identification of a functional regulatory element in this region is consistent with the published data.

It is important to note that the activity measured for each individual fragment resulted from independent oligo synthesis, cloning, sorting and sequencing. Therefore, we believe that obtaining positive activity for three consecutive fragments that contain the GNRA tetraloop by chance are very unlikely.

Another point was raised with respect to the similar activity levels obtained for one of the tested fragments and the full-length EMCV IRES. While there is no doubt that the studied structural conformation of the EMCV IRES could not be captured with a 174nt-long fragment, we cannot determine whether the full-length IRES was indeed in such conformation in the cellular environment of H1299 lung carcinoma cells used in our assay. It was shown that IRES-directed translation is cell line-dependent and that differences in the availability of *trans*-acting factors lead to various activity levels. One such study that was conducted by Martin Holcik, whose group assayed the activity of several IRESs, including the EMCV IRES, in six different cell lines and showed that their activities differ between cell lines (Nevins *et al.* JBC. 2003 (*8*)). Specifically, the EMCV IRES activity ranged from 80- to 350-fold relative to a control in 293T and T24 cells, respectively. Since to our knowledge there is no *in-vivo* structural data available for the EMCV IRES in H1299 cells, we cannot tell what is the secondary structure leading to the obtained activity of the full-length IRES. Thus, it is possible that its activity in these cells is mostly influenced by the GNRA tetraloop and that this activity was successfully captured with smaller fragments.

Investigating viral IRESs with oligos in the length of 174nt

Our technology, which is currently limited to sequences of length 174nt, does not allow detection and characterization of long viral IRESs as we clearly state in the manuscript and show specifically for the HCV IRES. However, some of the viral IRESs reported in the literature are in the range of ~200nt and were successfully captured in our assay. Among these IRESs are CrPV IGR (192nt), DCV IRG (189nt), gypsyD5 (261nt), HiPV IGRpred (188nt), TMV UI (231nt), HoCV1 IGRpred (188nt), and IAPV IRGpred (199nt). Thus, testing viral fragment in the length of 174nt is useful for the investigation of some viral IRESs but not all, such as viral IRESs with complex secondary structures.

All being said, it is important to clarify that high-throughput studies examining thousands of sequences cannot provide 100% certainty for each individual sequence tested. Our aim was to conduct the most rigorous controls possible for a library of 55,000 oligos, to provide the largest dataset of human and viral sequences for the scientific community, and to investigate the principles governing IRES activity through systematic analyses of a large number of sequences. In order to achieve 100% certainty at the single sequence level one should perform precise and laborious experiments which are not realistic in the case of high-throughput assays. We believe that combining insights from low- and high-throughput studies are essential to increase our understanding of IRESs specifically and translational control in general.

References

1. S. Weingarten-Gabbay *et al.*, Comparative genetics. Systematic discovery of cap-independent translation sequences in human and viral genomes. *Science* **351**, (2016).
2. S. Pyronnet, L. Pradayrol, N. Sonenberg, A cell cycle-dependent internal ribosome entry site. *Molecular cell* **5**, 607-616 (2000).
3. A. Pozner *et al.*, Transcription-coupled translation control of AML1/RUNX1 is mediated by cap- and internal ribosome entry site-dependent mechanisms. *Molecular and cellular biology* **20**, 2297-2307 (2000).
4. X. Zhang, C. Richie, R. J. Legerski, Translation of hSNM1 is mediated by an internal ribosome entry site that upregulates expression during mitosis. *DNA repair* **1**, 379-390 (2002).
5. M. Bakhshesh *et al.*, The picornavirus avian encephalomyelitis virus possesses a hepatitis C virus-like internal ribosome entry site element. *Journal of virology* **82**, 1993-2003 (2008).
6. M. Majumder *et al.*, The hnRNA-binding proteins hnRNP L and PTB are required for efficient translation of the Cat-1 arginine/lysine transporter mRNA during amino acid starvation. *Molecular and cellular biology* **29**, 2899-2912 (2009).
7. M. E. Robertson, R. A. Seamons, G. J. Belsham, A selection system for functional internal ribosome entry site (IRES) elements: analysis of the requirement for a conserved GNRA tetraloop in the encephalomyocarditis virus IRES. *Rna* **5**, 1167-1179 (1999).
8. T. A. Nevins, Z. M. Harder, R. G. Korneluk, M. Holcik, Distinct regulation of internal ribosome entry site-mediated translation following cellular stress is mediated by

apoptotic fragments of eIF4G translation initiation factor family members eIF4GI and p97/DAP5/NAT1. *The Journal of biological chemistry* **278**, 3572-3579 (2003).
