# Supplementary figures and images for: Sequence features of viral and human Internal Ribosome Entry Sites predictive of their activity
Source: PLoS Comput Biol. 2017 Sep 18;13(9):e1005734. doi: 10.1371/journal.pcbi.1005734 (PMC5630158; doi:10.1371/journal.pcbi.1005734)

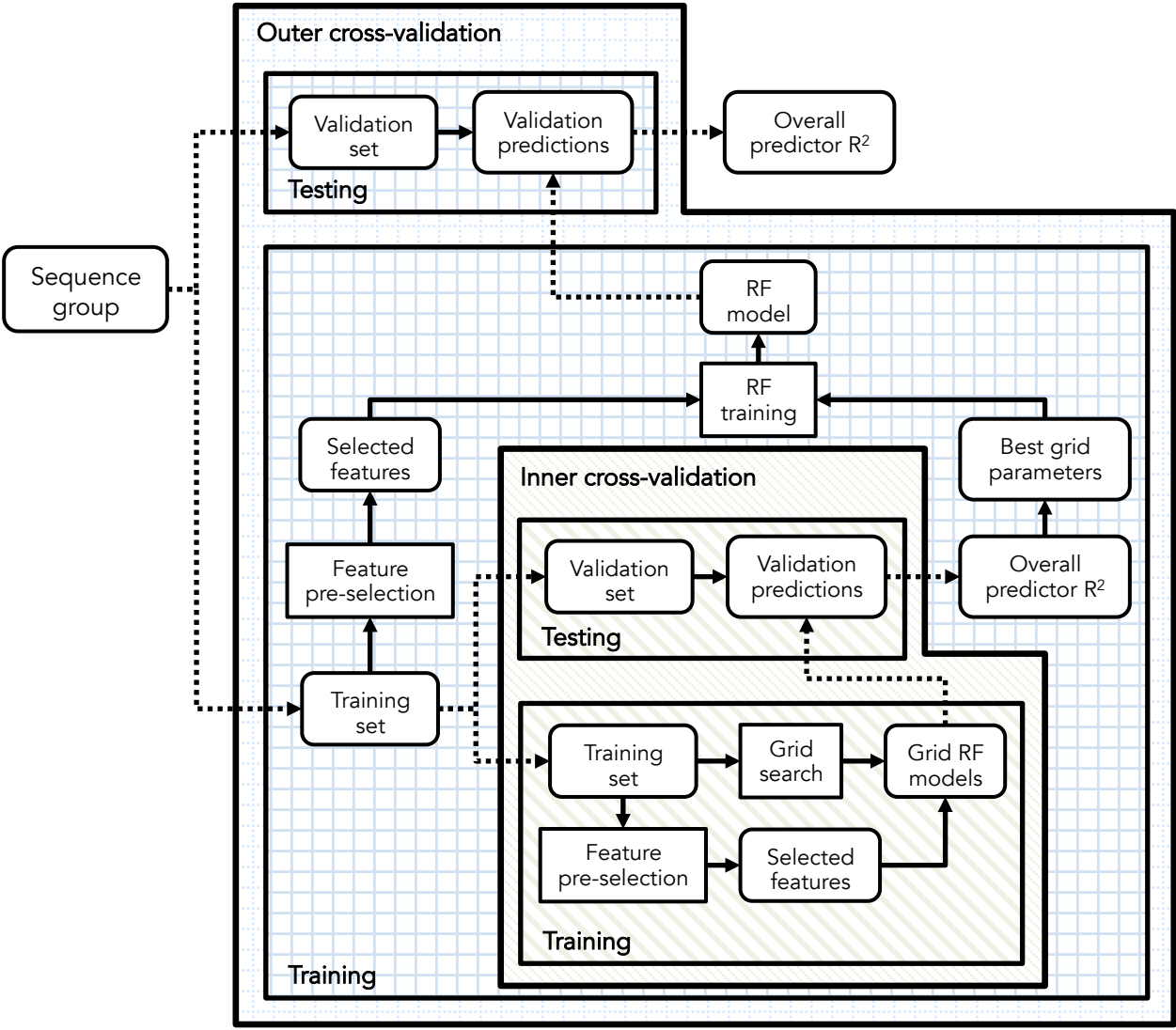

Supplement: S1 Fig — Inset plot shows distribution of IRES activity in active sequences (IRES activity above background levels). (PDF) [file pcbi.1005734.s003.pdf]

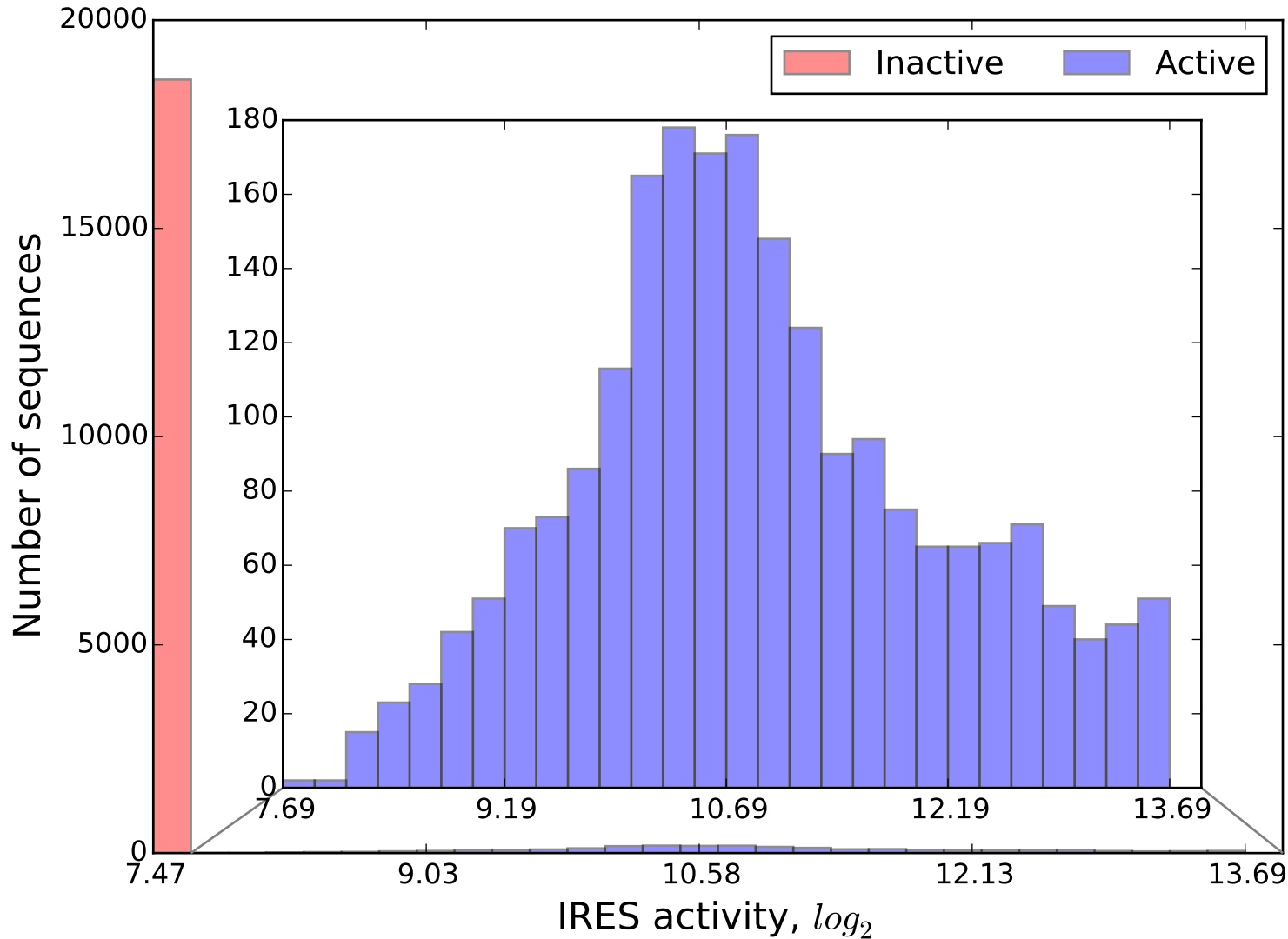

Supplement: S2 Fig — Rectangular boxes denote actions or procedures, whereas round boxes are used denote their input or output (results); hatched boxes group items that belong to the same CV loop (outer or inner) or CV set (training or testing); arrows show how information flows through the CV procedure, with the arrows crossing CV loop/set boundaries drawn using dashed lines. (PDF) [file pcbi.1005734.s004.pdf]

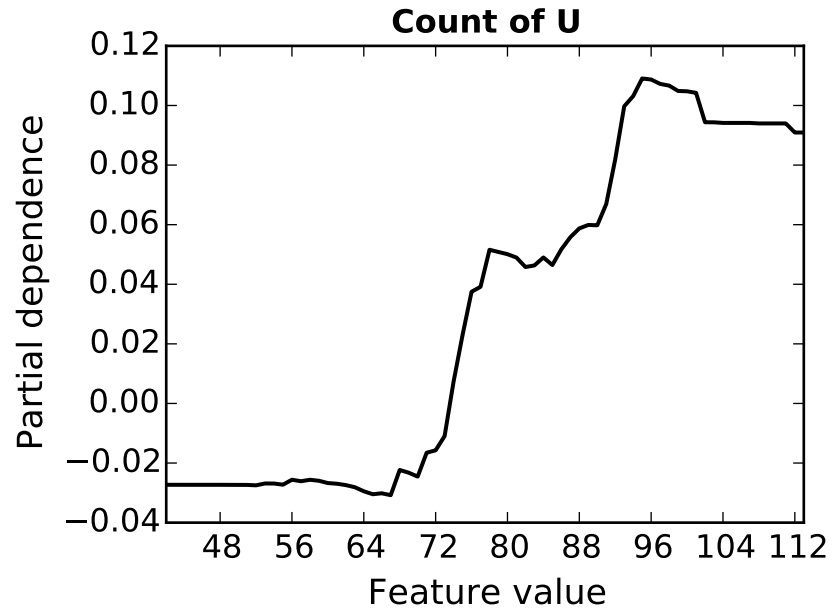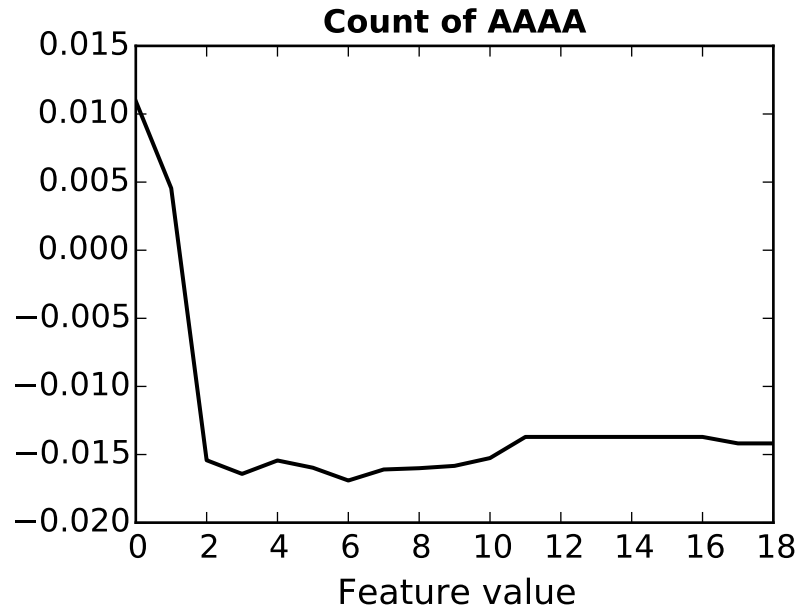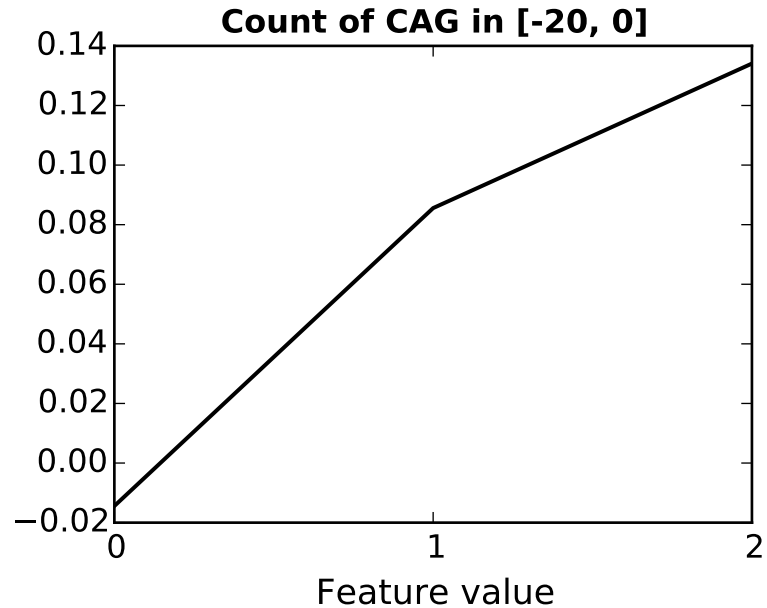

Supplement: S3 Fig — Three features from the dsRNA viruses models (k = 4, averaged over 10 CV folds): features U, AAAA and CAG in [−20, 0] (as shown in the order from left to right) were respectively classified as positive, negative and positive. (PDF) [file pcbi.1005734.s005.pdf]

**Human CDS**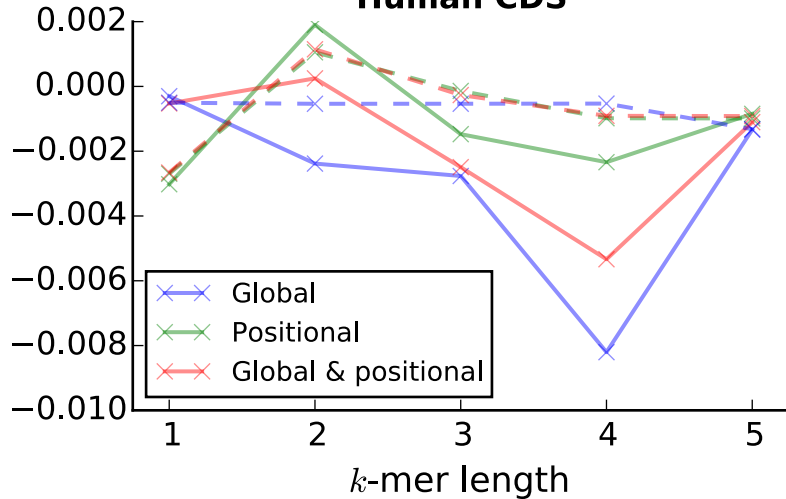**(-) ssRNA viruses**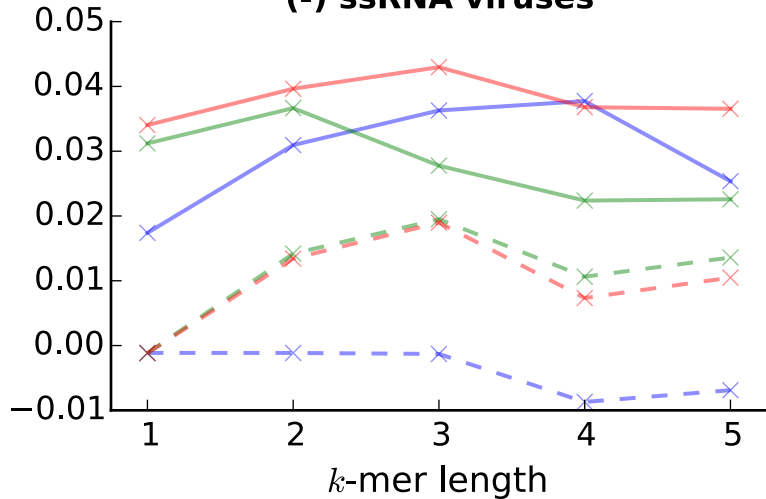

Supplement: S4 Fig — (PDF) [file pcbi.1005734.s006.pdf]

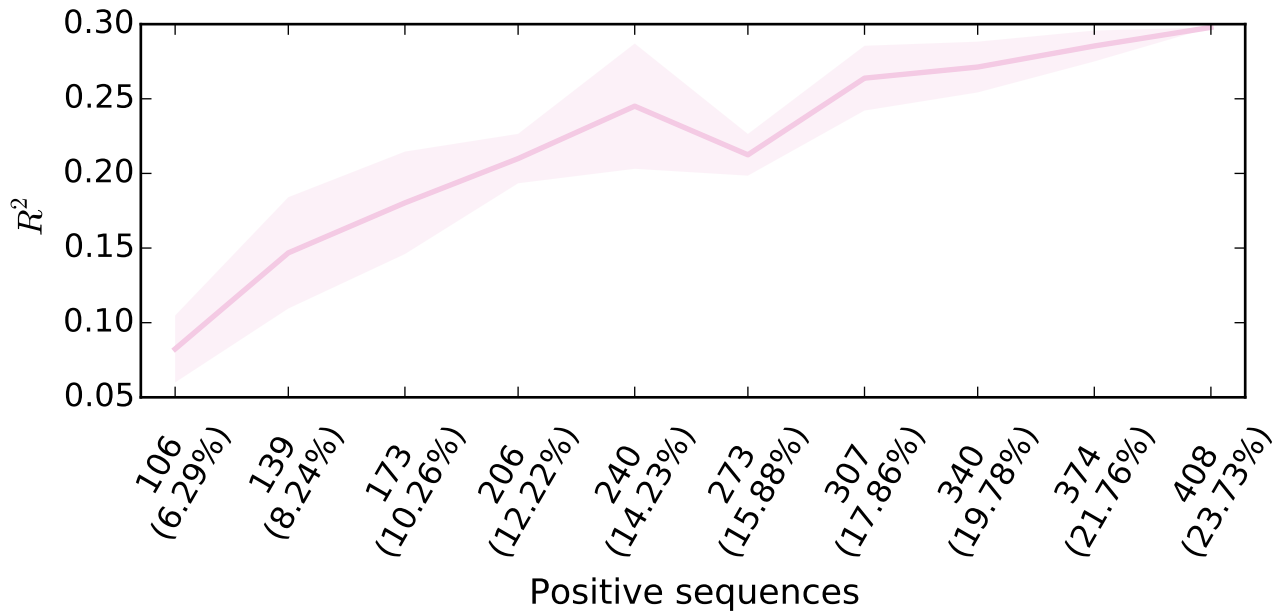

Supplement: S5 Fig — All models use global and positional k-mer counts (k = 4). Horizontal axis shows the number and the relative percentage of positive IRESs in the dataset, with the leftmost point (106 sequences) corresponding to the relative incidence of positive IRESs in the (−) ssRNA viruses group. Mean performance (solid line) and its standard deviation (shaded area) are shown for 5 random subsamples. These results indicate that small numbers of positive IRESs in a training set can limit predictive power of models trained on that set. (PDF) [file pcbi.1005734.s007.pdf]

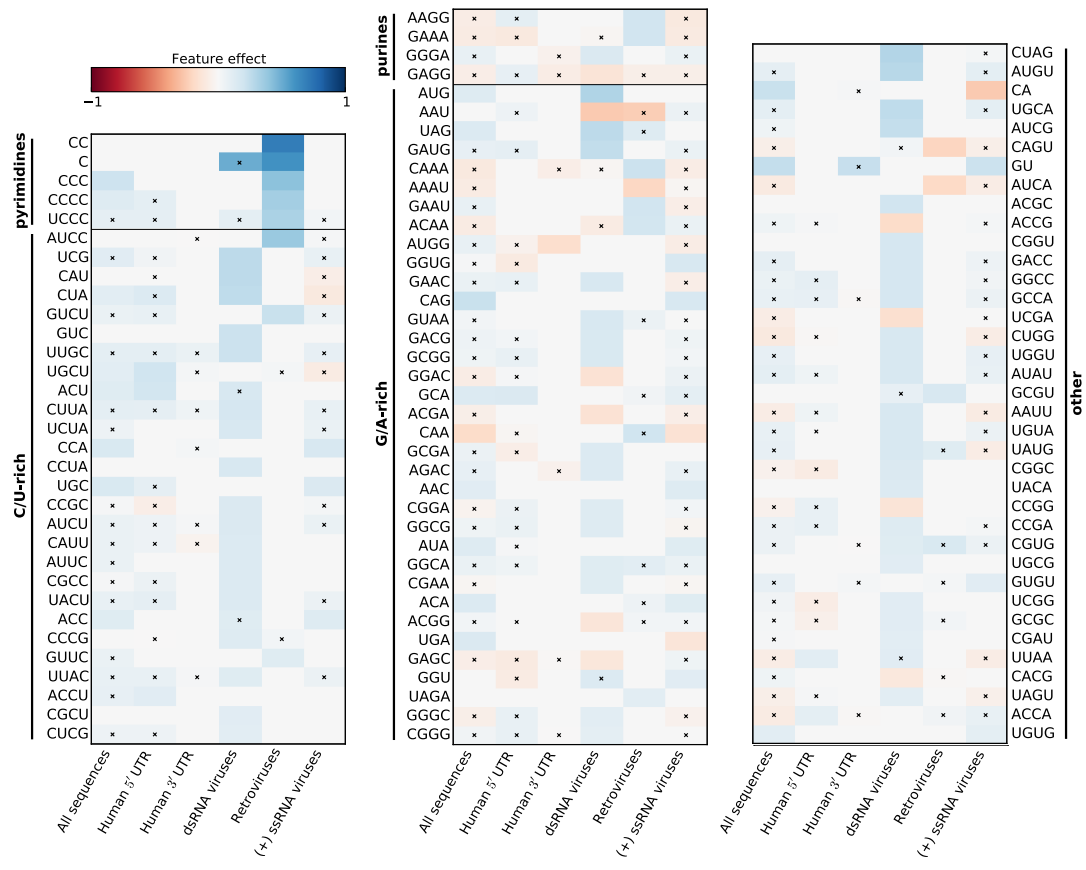

Supplement: S6 Fig — (PDF) [file pcbi.1005734.s008.pdf]

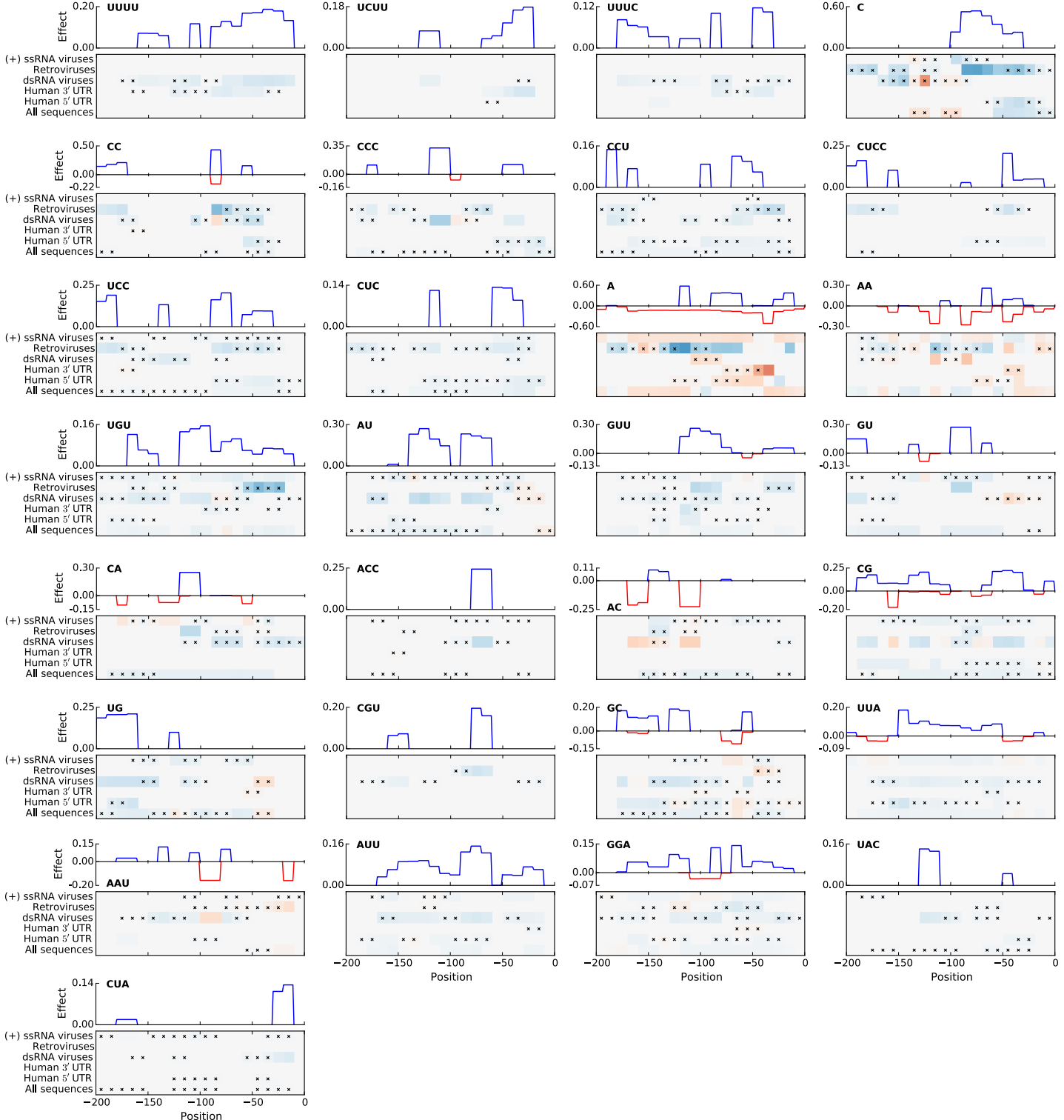

Supplement: S7 Fig — (PDF) [file pcbi.1005734.s009.pdf]

**A**      **Spacer 1 background (raw measurements)**

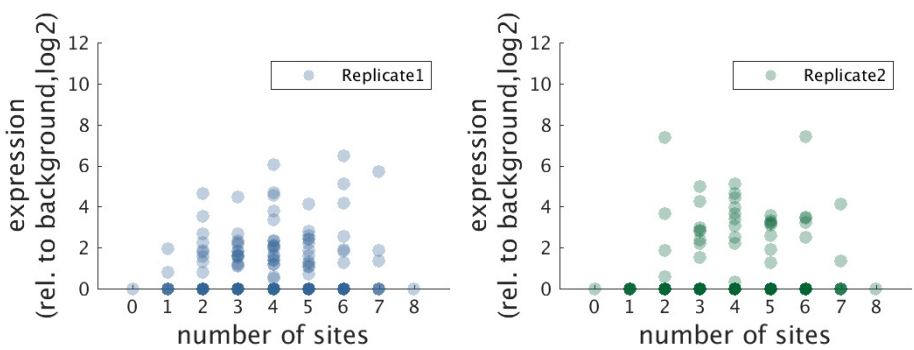

**B**      **HBB background (raw measurements)**

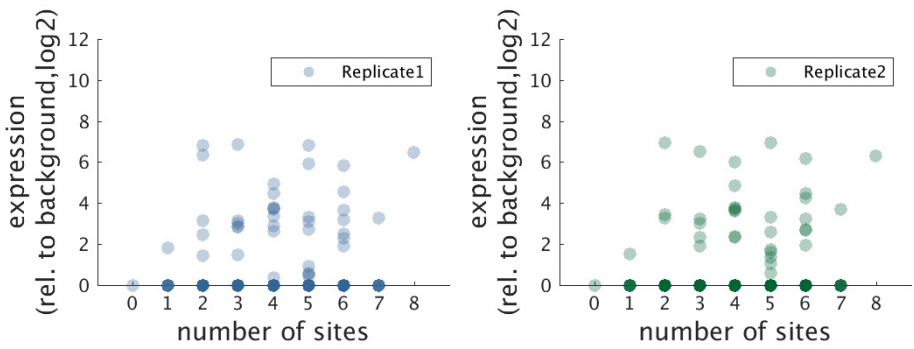

**C**

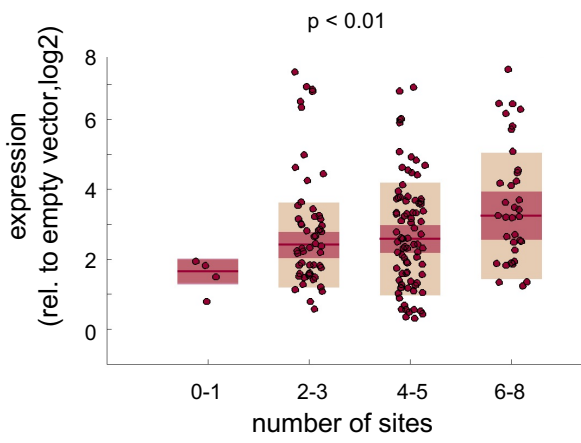

Supplement: S8 Fig — eGFP expression measurements of all the 512 designed oligos with 1-8 copies of the TEV IRES element (A) when placed in a synthetic background and (B) a native background from the human beta-globin (HBB) gene. (C) Joint analysis of the two backgrounds and the two biological replicates. Data was binned into four groups according to TEV sites number and one-way ANOVA was performed to determine if the difference between expression levels of the four bins is significant (p < 0.003). (PDF) [file pcbi.1005734.s010.pdf]

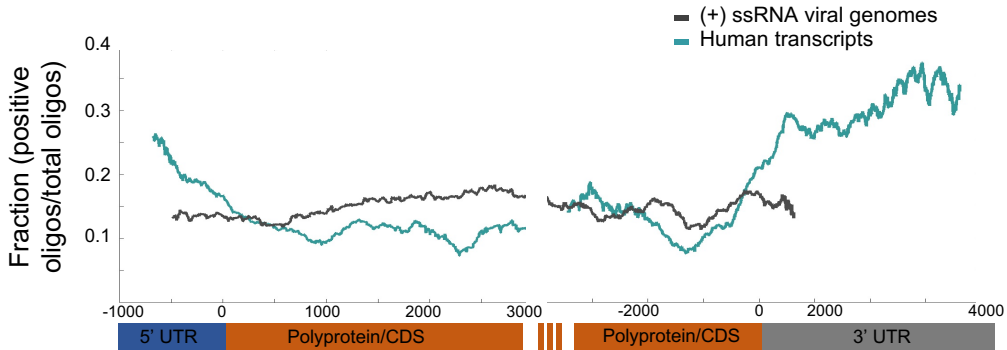

Supplement: S9 Fig — Moving average analysis of the fraction of positive IRESs across the 5′ UTR, coding sequence and the 3′ UTRs of human transcripts and (+) ssRNA viruses encoding a single polyprotein. In contrast to viral transcripts, which present uniform activity level across different regions, different activity level is obtained for human 5′ UTRs, coding sequences and the 3′ UTRs. (PDF) [file pcbi.1005734.s011.pdf]
