# Supplementary material for: Sequence features of viral and human Internal Ribosome Entry Sites predictive of their activity
Source: PLoS Comput Biol. 2017 Sep 18;13(9):e1005734. doi: 10.1371/journal.pcbi.1005734 (PMC5630158; doi:10.1371/journal.pcbi.1005734)
Supplement: S1 Table — (PDF) [file pcbi.1005734.s012.pdf]

| Name | Sequence |
|---|---|
| HBB | CTGACACAACTGTGTTCACTAGCAACCTCAAACAGACACCATGGTGCATCTGACTCCTGAGGAGAAGTCTGCCGTTACTGCCCTGTGGGGCAAGGTGAACGTGGATGAAGTTGGTGGTGAGGCCCTGGGCAGGCTGCTGGTGGTCTACCCTTGGACCCAGAGGT |
| Spacer1 | TCTGACATTTCTGACATTTCTGACATTTCTGACATTTCTGACATTTCTGACATTTCTGACATTTCTGACATTTCTGACATTTCTGACATTTCTGACATTTCTGACATTTCTGACATTTCTGACATTTCTGACATTTCTGACATTTCTGACATTTC |
